# A pilot randomized controlled trial comparing a novel compassion and metacognition approach for schizotypal personality disorder with a combination of cognitive therapy and psychopharmacological treatment

**DOI:** 10.1186/s12888-023-04610-5

**Published:** 2023-02-20

**Authors:** Simone Cheli, Veronica Cavalletti, Paul H. Lysaker, Giancarlo Dimaggio, Nicola Petrocchi, Francesca Chiarello, Consuelo Enzo, Francesco Velicogna, Francesco Mancini, Gil Goldzweig

**Affiliations:** 1 School of Human Health Sciences, University of Florence, Florence, Italy; 2 Center for Psychology and Health (Centro Di Psicologia e Psicoterapia), Tages Charity (Tages Onlus), Via Della Torretta 14, 50137 Florence, Italy; 3 Department of Psychiatry, Richard L Roudebush VA Medical Center, Indianapolis, USA; 4 Department of Psychiatry, Indiana University School of Medicine, Indianapolis, USA; 5 Centro Di Terapia Metacognitiva Interpersonale, Rome, Italy; 6 John Cabot University, Rome, Italy; 7 Guglielmo Marconi University, Rome, Italy; 8 The Academic College of Tel Aviv Yaffo, Tel Aviv, Israel

**Keywords:** Compassion, Evolution, Evolutionary systems therapy for schizotypy, Metacognition, Schizotypal personality disorder, Schizotypy

## Abstract

**Background:**

Schizotypal personality disorder is characterized by a pervasive pattern of maladaptive behavior that has been associated with the liability for schizophrenia. Little is known about effective psychosocial interventions. This pilot non-inferiority randomized controlled trial aimed to compare a novel form of psychotherapy tailored for this disorder and a combination of cognitive therapy and psychopharmacological treatment. The former treatment – namely, Evolutionary Systems Therapy for Schizotypy—integrated evolutionary, metacognitively oriented, and compassion focused approaches.

**Methods:**

Thirty-three participants were assessed for eligibility, twenty-four randomized on a 1:1 ratio, nineteen included in the final analysis. The treatments lasted 6 months (24 sessions). The primary outcome was change across nine measurements in personality pathology, the secondary outcomes were remission from diagnosis and pre-post changes in general symptomatology and metacognition.

**Results:**

Primary outcome suggested a non-inferiority of the experimental treatment in respect to control condition. Secondary outcomes reported mixed results. There was no significant difference in terms of remission, but experimental treatment showed a larger reduction of general symptomatology (η^2^ = 0.558) and a larger increase in metacognition (η^2^ = 0.734).

**Conclusions:**

This pilot study reported promising results about the effectiveness of the proposed novel approach. A confirmatory trial on large sample size is needed to provide evidence about relative effectiveness of the two treatment conditions.

**Trial registration:**

ClinicalTrials.gov; NCT04764708; Registration day 21/02/2021.

**Supplementary Information:**

The online version contains supplementary material available at 10.1186/s12888-023-04610-5.

## Background

Schizotypal personality disorder (SPD) reflects a pervasive and heterogeneous pattern of maladaptive experiences, which has been alternatively considered a personality disorder (PD), a subclinical form of schizophrenia or an expression of the liability for schizophrenia-spectrum psychopathology, often referred to as schizotypy [1]. It has been suggested that SPD (and schizotypy in the general population) may involve three facets [2, 3]. These would include positive features (e.g. ideas of reference, magical thinking, unusual experiences, paranoia), negative or interpersonal features (e.g. excessive social anxiety, anhedonia, lack of social interest), and disorganized features (e.g. odd thinking, speech and behavior, inappropriate or impulsive affect). Although SPD and schizotypy are separated constructs, they are associated with the same features: schizotypy would refer to a continuum between normality and even severe forms of psychopathology (i.e. schizophrenia), while SPD would be a highly representative clinical manifestation of schizotypy [4].

More recently, several scholars have posited how the conceptualization of SPD (and schizotypy) could benefit from a dimensional perspective, for example adopting the Five-Factor Model (FFM) and the Alternative Model of Personality Disorders (AMPD) [5–7]. From an FFM perspective, individual differences in SPD can be understood in terms of high levels of openness to experience (for example, being curious and open with respect to the facets of fantasy, actions, ideas), as well as some facets of low extraversion (warmth, gregariousness, and positive emotions), high neuroticism (anxiousness and self-consciousness), and low agreeableness (trust). According to AMPD, SPD consists of three facets from the domain of psychoticism (analogous to high openness) and three facets from the domain of detachment (analogous to low extraversion).

In spite of its prevalence ranging from 0.6% in a Norwegian non-clinical sample to 4.6% in an American non-clinical sample [8], little is known about effective psychosocial interventions for those diagnosed with SPD. A systematic review identified only three clinical studies [9] which reported SPD as a diagnosis, including two single cases and one randomized controlled trial (RCT). In the single cases SPD was a comorbidity, whereas in the RCT SPD was the primary diagnosis. The RCT described how a 2-year integrated intervention, which included different psychosocial interventions, such as assertive and social skills training for the patients and psychosocial education for patients and their families, reduced the risk for transition to psychotic disorder compared to a standard treatment which in most cases did not include any psychosocial interventions [10]. Of note, these studies did not target specific aspects of SPD psychopathology.

### Preliminary evidence for a compassion and metacognition therapy

After the publication of this review, Cheli and colleagues [11, 12] conducted two case series (*n* = 2; *n* = 12) in which they identified poor metacognition as a target for specifically tailored interventions that aim to improve metacognitive functioning.

Metacognition has been demonstrated to be related to symptoms and interpersonal problems in both personality disorders [13] and psychosis [14, 15] and to predict psychosocial functioning and severity of psychopathology in those diagnosed with personality disorders and schizophrenia spectrum and other psychotic disorders [16, 17].

As introduced above, the two cases series which targeted metacognition yielded promising results. The approaches adopted included either Metacognitive Reflection and Insight Therapy [18] (MERIT) which focuses on psychosis and Metacognitive Interpersonal Therapy [19, 20] (MIT) which is based on a specific case formulation for personality disorders. MERIT is an integrative recovery-oriented therapy aimed at stimulating a client's understanding of self and others by targeting interventions to the specific level of metacognition that the client is capable of within a single session or interaction, whereas MIT is a third wave therapy aimed at becoming aware and changing maladaptive interpersonal schemas and promoting healthy self-aspects. Preliminary evidence to date supported the effectiveness of both approaches on many outcomes, including the capacity to increase metacognition [21, 22].

At the same time, Cheli and colleagues [23–25] explored the interaction between poor metacognition and criticisms about self and others in those manifesting schizotypal features. It has been suggested how processes such as self-criticism and interpersonal criticism may trigger a threat response, characterized by reduced heart rate variability and hypo-activation of the prefrontal cortex allowing (not causing) the activation of the amygdala. Psychologically speaking, this would imply a reduced sense of safeness, with limited access to metacognition and difficulties in regulating intense feelings such as fear and anger once perceiving a threat [26]. In those struggling with schizotypy the social monitoring system may play a pivotal role in hyperactivating threatening beliefs about self and others [27]. This is consistent with several studies showing how self-criticism and blocks in receiving compassion from oneself and others are recurrent processes among several forms of psychopathology and, specifically, in those related to schizotypal features [28, 29].

Therefore, it has been hypothesized that an integrative form of psychotherapy consisting of a metacognitively oriented intervention and Compassion Focused Therapy [30] (CFT) would be beneficial for those diagnosed with SPD. Indeed, CFT is an evolutionarily informed, bio-psycho-social psychotherapeutic approach aimed at strengthening the capacity to soothe one’s own suffering and promote healthy forms of prosociality via the activation of a compassion motivation towards ourselves and others. CFT posits that the soothing system, a care-based mammalian affect regulation system, is poorly accessible in those struggling with either internal (e.g. self-criticism) or external (e.g. interpersonal criticism) threats. Those persons would have instead a hyperactivation of the threat detection and response system and difficulty in receiving compassion in the form of both self-compassion and compassion from others. This reduced ability to access affiliation-based motivation such as compassion, would compromise the possibility to experience a felt sense of inner safeness and the regulating activation of the prefrontal cortex, which makes it more difficult for individuals to fully access their metacognitive abilities [31].

Therefore, an integration of MERIT and CFT was preliminarily tested as either subsequent modules [23] or within the same synergistic format [32]. In both cases series (*n* = 6; *n* = 2) authors reported promising findings in treating persons with prominent schizotypal features. Although the sample size was small, this integrative form of therapy has proven effective with patients with negative, positive and disorganized facets of schizotypy. This preliminary evidence together with existing knowledge about the role of metacognition and self-soothing in severe mental disorders such as schizophrenia and PDs supported our integrative model. We acknowledge that several mechanisms and processes have been investigated in those with schizotypal traits and there are currently no guidelines for the treatment of SPD, particularly in terms of talk therapy. In this paper we present the results of a pilot RCT aimed at testing an implemented version of our new integrative intervention, that is the experimental treatment, namely Evolutionary Systems Therapy for Schizotypy (EST). This novel approach tries to follow Theodore Millon’s [33] suggestion about integration in psychotherapy. First, integration inheres the client not the therapist: The protocol is tailored on client’s features and personality. Second, integration has to be synergistic, that is it has not to simply include different approaches, but rather make them consistently interact with one another. Therefore, we propose that an evolutionary framework aimed at synergistically integrating CFT and metacognitively oriented approaches would effectively target the presumably core mechanisms of schizotypy: poor ability to experience interpersonal and intrapersonal safeness (i.e., underdeveloped soothing system) and concomitant poor ability about thinking, both with regard to their own thinking and the thinking of others (i.e. underdeveloped metacognition). Evolutionarily speaking, these two mechanisms are related to the rise (at both phylogenetic and ontogenetic level) of the human social brain [30, 34, 35]. Several studies suggest how schizotypy would be specific of our species and directly linked with the demanding complexity of human social brain [36]. Moreover, the core feature of schizotypy and SPD that is the proneness to experience odd behaviors, thoughts, and emotions would represent a maladaptive manifestation of one of the five personality traits—namely openness to experience – associated in turn with the capacity to mentalize and creatively master human challenges [5, 37]. Thus, we hypothesize that those with prominent schizotypal traits would experience increasing difficulty when confronted with perceived normative and judgmental stances from the surrounding social environment and self-critical beliefs in relation to their oddity [38]. This process would foster the well-known vicious cycle between perceived threat, reduced intra-personal safeness, poor metacognition, and difficulty in regulating negative feelings [26]. Thus, we hypothesize that an evolved social mentality such as compassion would support and strengthen the application of any technique aimed at promoting metacognition. Indeed, a compassion focused orientation should inform the very therapeutic relationship: a compassion focused stance of the therapist (i.e., intention, attitude, voice, posture, specific practices, etc.) are crucial in stimulating client’s metacognition as they might intrinsically nudge the patient to gradually adopt such a compassionate stance in looking at themselves and their suffering.

Thus, we suggest to support patients diagnosed with SPD by promoting metacognition and the capacity to soothe one’s own suffering at both subjective (self-to-self schemas) and intersubjective (self-to-other schemas) levels. By assuming a unified epistemological framework, we refer to these two levels in terms of system: A set of interacting elements that result in an emergent and integrated whole [39]. A detailed description of the protocol is reported in Methods.

### Research objective and hypothesis

The aim of this registered research (ClinicalTrials.gov; registration number NCT04764708; registration day 21/02/2021) was to preliminarily explore the effectiveness of a novel integrative recovery-oriented form of psychotherapy specifically tailored to clinical manifestations of SPD (i.e. ESTS). Thus, we pilot-tested through a parallel double-blinded RCT on repeated measures the non-inferiority between the experimental treatment and a combination of cognitive behavioral therapy (CBT) for PDs [40] and a mandatory personalized psychopharmacological treatment based on existing guidelines for SPD [9]. On the one hand, ESTS is a evolutionarily oriented therapy that integrates MOP and CFT techniques. It has been specifically tailored on schizotypal traits and posits how these traits would represent a maladaptive manifestation of high openness to experience and low extraversion. On the other hand, CBT for PDs is therapy based on the standard cognitive model that suggests how PD is maintained by a combination of maladaptive beliefs about self and others, and contextual factors that reinforce problematic behavior and undermine effective behavior.

Recruited patients were allocated on a 1:1 ratio between the two arms. Our hypothesis was that the experimental treatment without medication would show effectiveness at least equal to a presumably effective treatment that integrated CBT and psychopharmacological interventions (registered primary and secondary outcomes are described in Methods). This hypothesis is consistent with studies that report how interventions tailored on the specific clinical manifestations of psychosis can be as feasible as a combination of psychosocial and psychopharmacological interventions [41]. Moreover, we hypothesized how a therapy specifically tailored on SPD such as ESTS would achieve outcomes at least equal to a good treatment as usual integrating medication and CBT. Finally, the results of this pilot RCT should allow the development (e.g. sample size calculation; treatment adherence scale definition; etc.) of a subsequent confirmatory RCT.

## Methods

### Participants

#### Patients

**Fig. 1:**
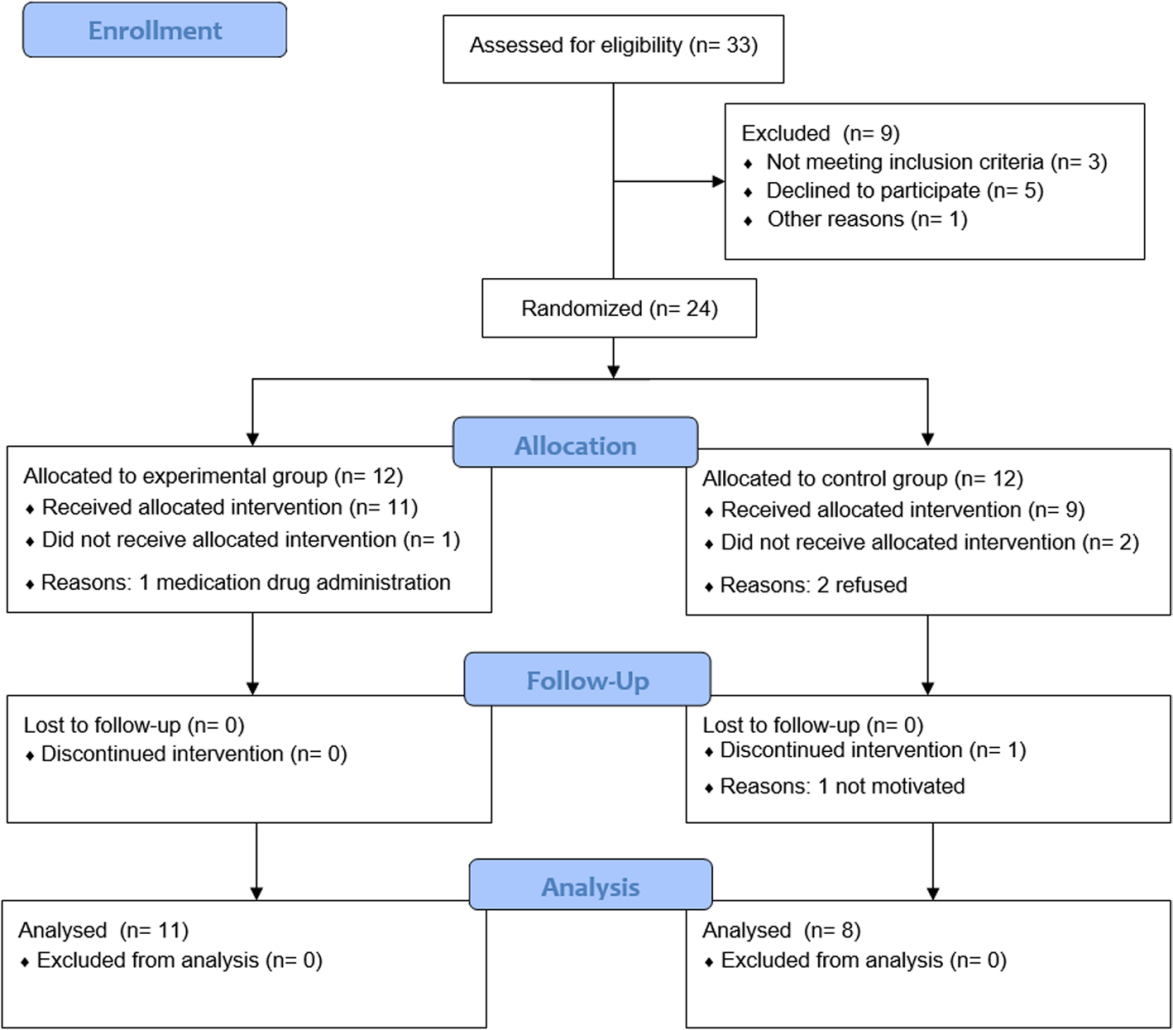
Flow diagram of the randomized controlled trial

Registered sample size calculation (f = 0.33; α = 0.05; 1-β = 0.95; groups = 2; measurements = 9) suggested a minimum size equal to 14 (critical F = 2.036) with an actual power equal to 0.9704. By estimating a drop-out rate of 20% (*n* = 2.8) we recruited participants until the end of the scheduled time frame (1 year) to reach a sample bigger than 17 (allocation ratio 1:1). Inclusion criteria were being (a) at least 18 years old, (b) diagnosed with SPD, and (c) able to read and sign the informed consent form. Exclusion criteria were being (a) under any psychosocial or psychopharmacological treatment, (b) diagnosed with one or more of the following: schizophrenia and other psychotic disorders and/or bipolar disorder and/or intellectual disability and/or any neurological disease. Of 33 persons assessed for eligibility, 24 were randomized, 19 included in the final analysis (see Fig. 1). Most of the sample of randomized patients was made up of men (62.5%) with a high school degree (54.2%) and a low-middle family income (50%); the average age was 24.75 years (see Table 1).

**Table 1:** Socio-anagraphic data

	Control Group		Experimental Group		Total Sample	
	n	%	n	%	n	%
Sex						
Male	8	66.7	7	58.3	15	62.5
Female	4	33.3	5	41.7	9	37.5
Education						
Middle school or less	1	8.3	1	8.3	2	8.3
High school	7	58.3	6	50	13	54.2
College	4	33.3	4	33.3	7	58.3
Advanced degree	1	8.3	1	8.3	2	8.3
Annual family income						
15.000–35.000€	2	16.7	2	16.7	4	33.3
35.000–70.000€	6	50	6	50	12	50
> 70.000€	4	33.3	4	33.3	8	33.3
Undesired events^a^						
Drop-out	3		1		4	
Psychotic episode	0		1		1	
Self-injuring behavior	2		1		3	
Drug side-effects	4		NA		NA	

*N* = 24 (*n* = 12 in each group). Participants mean age was 24.75 years old (*SD* = 3.33). Percentages of undesired events (a) are not reported since the same patients may have experienced more than one event. Drug side-effects are not reported for experimental group since medication was an exclusion criterion

#### Therapists

Therapists were included as participants in this study. Inclusion criteria for all the therapists were the following: (a) being 18 years or older, (b) being a clinical psychologists who had completed a 4-year formal psychotherapy training, (c) having at least 5 years of experience in treating PDs. More specifically, study therapists must have had at least 5 years of experience in either CBT or metacognitively oriented therapy plus CFT for control or experimental group, respectively. Therapists also had to have the (d) availability to provide psychotherapy to clients diagnosed with SPD, and (e) availability to engage in 90-min of supervision every two weeks. Considering the limited sample size of the RCT and the novelty of the experimental treatment, we opted for minimizing the variance of therapists skills and competences and maximizing the adherence to an ideal curriculum of a training program that has not been implemented yet. Therefore, we found just two therapists who could provide SPD-tailored psychotherapy, one for each arm of the study.

### Measures

The Metacognition Assessment Scale – Abbreviated [14] (MAS-A) is a scoring system for assessing metacognition. It comprises a total score (ranging from 0 to 28) and four subscales (self-reflectivity; understanding others’ minds; decentration; mastery). In accordance with best practices, the MAS-A was used in conjunction with the Indiana Psychiatric Illness Interview [42] (IPII) which is a semi-structured individual interview aimed at assessing illness narrative. Therefore, transcripts of IPII were coded through MAS-A. Inter-rater reliability of single scales has been show to vary between 0.68 and 0.98 [43, 44].

The Personality Inventory for DSM-5 – Brief Form [45] (PID-5-BF) is a brief reliable self-report (Cronbach’s α ranging between 0.89 and 0.91) aimed at assessing the five domains (negative affectivity, detachment, antagonism, disinhibition, psychoticism) of the DSM-5 Alternative Model of Personality Disorders (AMPD) using an aggregated score for the general personality pathology. In this study, only the total score was used.

The Structured Clinical Interview for DSM-5 Alternative Model of Personality Disorders [46] (SCID-5-AMPD) is a semi-structured diagnostic interview for PDs as defined by the dimensional model of the DSM-5. It comprises three different modules. In this study we used Module III that aims at comprehensively assessing each of the six specific personality disorders of the Alternative Model, SPD included. SCID-5-AMPD was used to confirm the SPD diagnosis.

The Symptoms Check List-90—Revised [47] (SCL-90-R) is a widely used and highly reliable self-report questionnaire (Cronbach’s α ranging between 0.74 and 0.96) for assessing several dimensions of psychopathology and obtaining an aggregated score of psychosocial distress (global severity index [GSI]). In this study, only the GSI was used.

### Procedures

As reported in the registered protocol, this study was approved by the Ethics Committee and Institutional Review Board of the first author (Reference Number 032020–07,072,020). The study was conducted in accordance with the Declaration of Helsinki and informed consent was obtained from all the participants (clients and therapists). Clients and therapists were recruited through emails and online posts advertising the study. After having signed informed consent form, therapists were assessed for eligibility by the researchers responsible for supervision (supervisors) and patients by the researcher responsible for assessment (assessor). All the clients completed as pre-assessment the (t_1_) SCID-5-AMPD, IPII, PID-5-BF, and SCL-90-R (see Measures). The same measures were assessed at 1-month follow-up (t_9_). At the beginning of the intervention (t_2_) and at the end of each of the 6 months of psychotherapy (t_3-8_) participants filled out the PID-5-BF only. The two therapists adhering to the inclusion criteria were assigned based on their CV to the two arms by the two supervisors of the control (CG) and experimental group (EG). Another researcher responsible for monitoring all the procedures (investigator) randomized the patients using a computer software [48]. As suggested by guidelines for RCT in psychotherapy [49] investigator double-blinded assessor and statistician (researcher responsible for analyzing the final database), rather than therapists and patients. Given the specificity of the interventions it would have been impossible to blind either patients or therapists, while blinding the researchers in charge of analyzing the outcomes was possible and useful in reducing biases of the study. Thus, neither the assessor nor statistician was aware of the allocation of the patients since the investigator anonymized all the data before sharing with them.

All randomized patients were supposed to attend a 6-months of weekly therapy (total expected sessions = 24). They did not receive any compensation for the study. EC therapist and CG therapist were supervised every two weeks (by EC supervisor and CG supervisor, respectively). Supervision lasted 90 min and followed a general outline focused on: (a) evaluation of the therapeutic alliance; (b) evaluation of therapist’s conceptualization; (c) evaluation of the therapist's adherence to the protocol; (d) evaluation of the treatment plan; (e) evaluation of changes in psychopathology; (f) and evaluation of patient's feedbacks and requests. In addition to supporting clinical work, the supervision aimed at confirming adherence to the specific intervention provided.

In the same week in which the first session of the CG took place, a psychiatrist (MD) completed an initial visit to set up medication in accordance with guidelines for SPD [9]. A follow-up visit was then carried out every month and paralleled by an update with the referral therapist during the CG supervision. The same psychiatrist participated in EG supervisions once a month to assess the severity of symptoms. The psychiatrist could schedule a follow-up visit with patients included in the EC and suggest medication drugs. In this case the patient would be excluded from the study, since the EG explored a medication-free treatment.

Supervisors were recruited if they had more than five years of experience in the specific treatment (CFT and MOP for EC supervisor; CBT for CG supervisor), whereas psychiatrists if they had more than five years of experience in schizophrenia and PDs.

In order to reduce allegiance bias, the statistician was affiliated with a different institution than the one of therapists and supervisors. In addition, the statistician, investigator and psychiatrist were selected as declaring themselves as from a different psychotherapy orientation in respect to the one of EG intervention.

At the end of the study (that is after the 1-month follow-up), the therapists and patients were asked to be willing to continue the therapy. Both therapists accepted, and the majority of patients continued therapy after the interruption between final and follow-up assessment (EG = 10; CG = 6). All the interventions were delivered at the Center for Psychology and Health, ANONYMIZED.

### Primary and secondary outcomes

Consistent with pre-registration, primary outcome was the difference between the two arms (EG, CG) in terms of changes in personality pathology (PID-5-BF total score) across the nine measurements (t_1-9_). Secondary outcomes were differences at follow-up assessment (t_2_) between the two arms in terms of (a) rate of remission from SPD diagnosis (SCID-5-AMPD), (b) pre-post changes (t_1_ vs t_9_) in general symptomatology (SCL-90-R total score), and (c) pre-post changes (t_1_ vs t_9_) in metacognition (MAS-A total score). The described outcomes were hypothesized to confirm the non-inferiority of the EG in respect to the CG.

### Treatment conditions

#### Control group intervention

The patients allocated to the CG completed a combination of CBT for PDs [40] and a mandatory psychopharmacological treatment in accordance with existing guidelines for SPD [9]. Since evidence based interventions for SPD are not reported, this combination was supposed to be an effective control treatment. CBT for PDs is an effective intervention based on a general model related to the PD clinical condition and integrates a combination of techniques—such as Socratic dialogue and cognitive restructuring—and psycho-education [50]. CBT for PDs is mainly focused on changing the core beliefs related to a specific client’s condition and promoting more functional mechanisms to cope with these beliefs. Patients diagnosed with SPD may see themselves as loner and different and the others as unfriendly and hostile, fueling overdeveloped strategies in the form of “putting on a mask”, “keeping distance”, and “cultivating unusual appearance”. Consequently, the intervention would promote undeveloped strategies such as developing and nurturing social connection. In CG, patients received psychopharmacological treatment on an individualized basis, with medication selected among the ones described in guidelines for SPD [9].

#### Experimental group intervention

The patients allocated to the EG attended a specifically designed intervention (see Table 2), labeled for the purpose of this study as ESTS. ESTS is an integrative, recovery-oriented therapy that aims at synergistically combining different approaches consistently with client’s schizotypal personality [33, 51]. Our experimental intervention focuses on an evolutionary look at human schemas, processes and automatisms as evolved psychosocial mechanisms rather than on categorical or normative approaches to diagnosis and psychopathology [52]. Suffering may come from repeatedly applying strategies that are maladaptive in respect to our healthy evolutionarily evolved motives and mentalities. For example, patients diagnosed with SPD may maximize their own oddity as a defensive, even maladaptive, strategy in response to a perceived judgmental stance from the social environment. This would lead to social disconnection and a reduced capacity to soothe their suffering and receive support from the others. This model is consistent with an understanding of personality pathology through a dimensional and interpersonal viewpoint [53]: SPD may feature both oddity and problems in socializing. Some of them, with minimal awareness of the reason for their social behavior, may opt for a defensive risk minimization strategy, that distances themselves from others because they fail to share their own worldview and understand the one of the other. As a consequence of this detached behavior, their chances to interact remain limited. Moreover, they may have an attention bias towards negative judgments from others about their bizarre beliefs, which is another reason leading them to reduce social contacts. Indeed, they may consider that the others are not able to understand them.

**Table 2:** Outline of Evolutionary Systems Therapy for Schizotypy

Phase	Goal	Psycho-education	Narrative techniques	Experiential techniques
*1) Sharing*	Sharing treatment goals and conceptualization	Social safeness and social cognition Oddity and detachment from an evolutionary viewpoint	Stimulating autobiographical memory Exploring therapeutic relationship	Grounding Soothing breath
*2) Subjective systems*	Experiencing mind–body awareness of subjective experience	Early maladaptive schemas Temperament and personality	Stimulating self-reflectivity and decentering Functional analysis of self-criticism	Realities of life meditation Compassionate self or image
*3) Intersubjective systems*	Experiencing mind–body awareness of intersubjective experience	Interpersonal schemas and cycles Compassion and prosociality	Stimulating awareness of the other Metacommunication	Compassionate imagery with rescripting Compassionate chairwork
*4) Consolidation*	Managing and consolidating changes	Psychosocial adaptation Error friendliness	Stimulating mastery Reflecting on changes	Individualized techniques Client’s workbook of practices

Experimental treatment—namely, Evolutionary Systems Therapy for Schizotypy—comprises four phases, partially overlapping each other. Each phase ranges between 4 to 8 sessions. Goals and psychoeducation were defined in accordance with the proposed integrative intervention and evolutionary framework. Narrative and experiential techniques were primarily derived from Metacognitively Oriented Psychotherapies and Compassion Focused Therapy, respectively. The contents of the four phases are indicative of priority elements of the interventions that can also interact between each other. For example, a technique from a later phase can be used in a previous one, and vice versa

According to this view, ESTS aims at promoting metacognition [15] and cultivating compassion towards ourselves and others [30]. Promoting metacognition is important in SPD because these individuals tend to display a sense of fragmented self-experience and the therapeutic techniques used are selected on the base of the in-session level of metacognitive functioning [14, 42]. In this respect, facing fragmentation from a metacognitive perspective means, for example, promoting self-reflectivity by stimulating autobiographical memories [54] and decentration through the insertion of the therapist’s mind in the therapeutic dialogue [18]. Another element of ESTS is a consistent focus on repairing fractures in intersubjectivity these persons suffer from. They may be guided by early maladaptive schemas related to attachment and, broadly, interpersonal experiences which drives poor social functioning; they are also likely to have problems in establishing connections with others to the point they experience distance, alienation and disconnection [19, 55–58].

As previously summarized, ESTS directly refers to an evolutionary perspective that is consistent with evidence suggesting how schizotypy emerged with the rise of our complex social brain [34, 36] and its clinical manifestations are worsened by reduced abilities to access care-based social mentalities, like compassion, with resulting hyperactivation of the threat system and hypoactivation of the soothing system [27, 30]. Patients are supported in understanding which experiences are related to these two different motives aimed at either defending oneself from perceived threat or soothing one’s own suffering and promoting social safeness. Several experiential techniques—such as soothing breathing, compassionate imagery, compassionate chairwork – are used to help the patient to use more adaptive strategies in looking at themselves and others [59].

During the early phases of the intervention the therapist shares a specific evolutionary conceptualization of SPD [38]: Client's traits—presumably high openness and low extraversion—may have knocked against a felt sense of normative or even aggressive responses from the surrounding environment, so triggering maladaptive patterns in the form of oddity and detachment. Early experience of bullying, for example, are frequently reported by patients as social responses to their odd and bizarre behaviors. These patterns, in turns, would have hindered client's metacognitive functioning and capacity to deactivate the threat system and soothe oneself. The more the patients perceive others as threatening, the more they use automatic defensive strategies (i.e., emotional and interpersonal detachment, and self-criticism) which reduce their inner sense of safeness, and the capacity to mentalize subjective and intersubjective experiences. Table 2 is indicative of how ESTS integrates an evolutionarily oriented psycho-education with narrative and experiential techniques in order to promote a transitioning from maladaptive forms of oddity and detachment toward an increased ability to experience compassion for self and others and appreciation of ones’ own unique personality.

### Statistical analysis

Differences in all the variables at baseline (t_1_) between the two hams (EC, CG) were preliminarily explored via one-way ANOVA and odd ratio (OR) for continuous and categorical variables, respectively. The primary outcome (PID-5-BF) was explored through General Linear Model with Repeated Measures (GLM-RMs). The secondary outcomes were analyzed through OR and GLM-RMs for categorical (remission from diagnosis) and continuous (SCL-90-R and MAS-A total scores) variables, respectively. By considering the exploratory nature of the RCT, we opted for an intention-to-treat analysis including all the drop-outs and missing data. All the analyses were performed through SPSS version 25. Main outcomes are ported in Results, whereas the complete SPSS outputs in Supplementary Materials together with some additional analyses.

## Results

Between December 20 2020 and September 30 2021 we recruited 33 patients, of these 9 (27.27%) were excluded due to either not meeting the inclusion criteria (3/33, 9.09%), declining to participate once asked to sign the informed consent form (5/33, 15.15%), or moving to another city (1/33, 3.03%). The remaining 24 patients were randomly allocated (see Fig. 1): 12 to EG and 12 to CG. Of these 4 (4/24, 16.66%) dropped out before the completion of the study. Of these 1 (1/12, 8.33%) allocated in EG (E3) was excluded because the study psychiatrist opted for introducing an antipsychotic medication after a severe brief psychotic episode (BPE) between session 4 and 5. Three (3/12, 25%) patients in the CG abandoned the study for different reasons: C3 and C10 reported that they were distressed by the treatment, including both the psychotherapy and the medication, and finally refused it (between session 9 and 11), C7 showed a discontinued adherence in terms of session attendance to the treatment, and dropped out after session 6.

Eight (8/24, 33.33%) persons reported unintended effects, including the four that dropped out. Four patients (including 2 that dropped out) in CG experienced low-to-moderate side effects during the antipsychotic medication (C3, C10, C2, and C9). One (C9) also reported intermittent self-injuring behaviors between the end of the first month and the beginning of the fifth month. Three other patients showed self-injuring behaviors in the first two months of the intervention, and then remitted, 1 from EG (E5) and 2 from CG (C1 and C8). As reported, one patient in the EG (E3) had a BPE and was excluded from the study to receive medication.

**Fig. 2:**
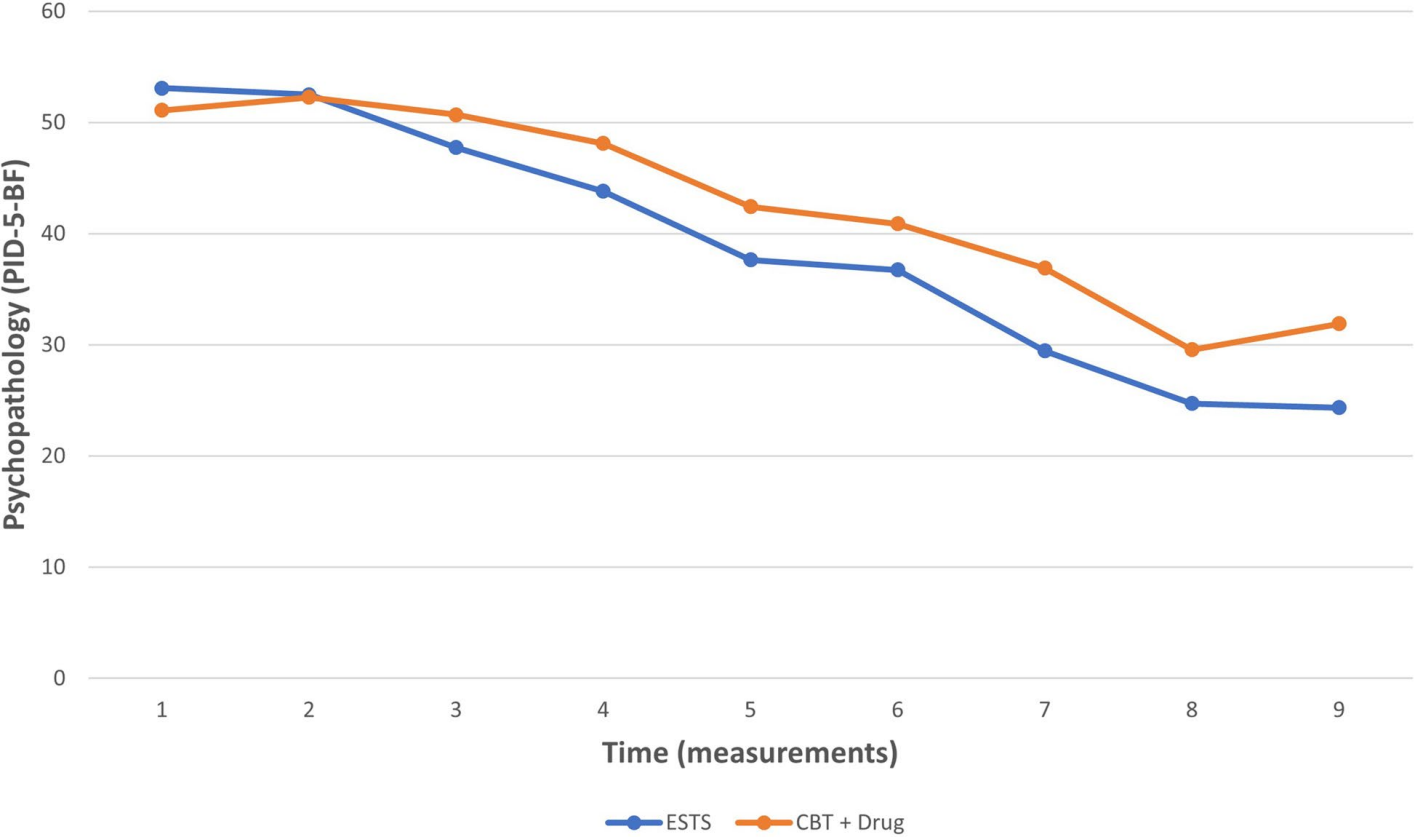
Mean personality pathology across measurements. *Note*. We report on y-axis mean scores at personality pathology (PID-5-BF) for those receiving either Cognitive Behavioral Therapy plus psychopharmacological treatment (CBT + Drug) in control group or Evolutionary Systems Therapy for Schizotypy (ESTS) in experimental group. The x-axis refers to the nine measurements (t_1-9_) of personality pathology as the primary outcome of the study

No significant differences between the two arms were found at baseline (t_1_) in any of the collected variables (see Supplementary Materials). GLM-RMs revealed that there was no statistically significant interaction between the effects of time and arm on personality pathology (F(8,144) = 1.935; *p* = 0.059; η^2^ = 0.097; C.I. = 95%), suggesting a non-superiority of CG (see Fig. 2 and Table 3). The same interaction showed three significant differences at within-subjects contrasts between initial assessment (t_1_) and single levels (t_2-9_): level t_3_ (F(1,18) = 6.514; *p* = 0.020; η^2^ = 0.266; C.I. = 95%), t_7_ (F(1,18) = 5.912;* p* = 0.026; η^2^ = 0.247; C.I. = 95%), and t_9_ (F(1,18) = 5.719; p = 0.28; η^2^ = 0.241; C.I. = 95%). These results may suggest a possible superiority of EG for these levels and non-superiority at the other levels, confirmed by a large effect size (η^2^ ranging between 0.241 and 0.247).

**Table 3:** Group-by-time interaction effect on primary outcome

	F (8,144)	P	η^2^
Personality pathology * Arms	1,935	,059	,097

We report the test (sphericity assumed) of within-subjects effects. Table 3 shows interaction between the effects of time (nine measurements, t_1-9_) and arm on personality pathology. Partial eta squared (η^2^) was computed using α = .05 (C.I. = 95%). Values higher than .14 are indicative of a large effect

**Fig. 3:**
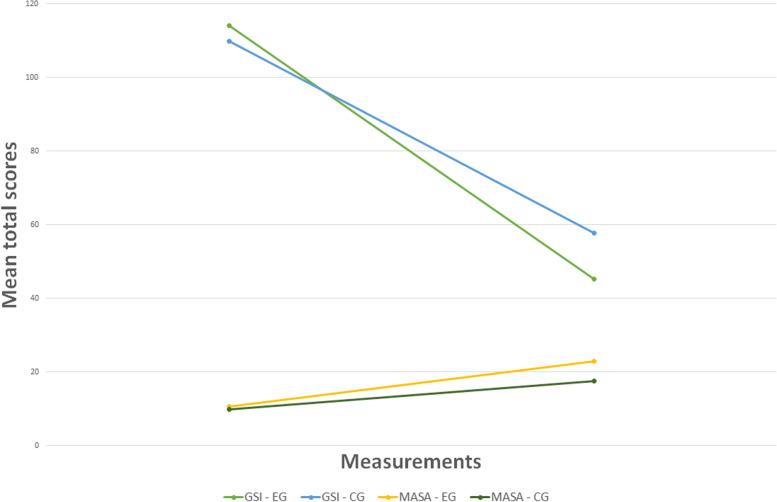
Changes in symptomatology and metacognition. *Note*. We report changes in mean scores of symptomatology (higher scores are indicative of more severe symptoms) and metacognition (higher scores are indicative of a better metacognitive functioning). The x-axis refers to the initial and final assessment. The y-axis refers to the mean total scores of the two measures: Symptoms Check List 90—Revised (SCL-90-R) and Metacognition Assessment Scale—Abbreviated (MAS-A). GSI-EG = mean total scores of SCL-90-R in experimental group; GSI-CG = mean total scores of SCL-90-R in control group; MASA-EG = mean total scores of MAS-A in experimental group; MASA-CG = mean total scores of MAS-A in control group

The secondary outcomes reported mixed results at 1-month follow-up. OR did not show significant differences in rate of remission from SPD between the two groups (OR = 3.667; C.I. = 0.323–41.590; χ^2^ = 1.2; *p* = 0.273), suggesting a non-superiority of any arm. GLM-RMs reported significant differences between EG and CG for both general symptomatology (η^2^ = 0.558; C.I. = 95%) and metacognition (η^2^ = 0.734; C.I. = 95%), suggesting a possible superiority of the experimental treatment (see Table 4 and Fig. 3) that is consistent with a very large effect size (η^2^ ranging between 0.558 and 0.734).

**Table 4:** Group-by-time interaction effect on secondary outcomes

	F (1,18)	P <	η^2^
General symptomatology * Arms	22.758	.000	.558
Metacognition * Arms	59.400	.000	.734

We report the test (sphericity assumed) of within-subjects effects. Tables 4 shows interaction between the effects of time (pre-assessment vs follow-up assessment, t_1_ vs t_9_) and arm on general symptomatology and metacognition, respectively. Partial eta squared (η^2^) was computed using α = .05 (C.I. = 95%). Values higher than .14 are indicative of a large effect

Finally, the whole sample showed a high rate of completion (83.33%) and significant differences between initial and follow-up assessment for all the measures (η^2^ ranging between 0.893 and 0.976; C.I. = 95%). No differences were found between arms in rate of drop-outs, suggesting a low attrition.

## Discussion

Our pilot, parallel, double-blinded RCT suggested how the proposed experimental intervention (i.e. ESTS) may be as effective as a combination of CBT and psychopharmacological treatment. At the initial assessment there were no differences between the two conditions across any of the variables investigated, and at 1-month follow-up we found no attrition. Both the interventions were possibly safe and feasible, showing a significant reduction of psychopathology and a high rate of completion and remission from SPD diagnosis.

The changes across time in the primary outcome (PID-5-BF) are seemingly indicative of non-inferiority of our specifically developed intervention—namely, ESTS—in respect to control group. Although the measure used (i.e. PID-5-BF) is a screening and not a diagnostic tool, the initial and final mean scores are consistent with scores above and below the mean of the normative sample, respectively. Therefore the pre-post changes may be indicative of a clinically significant reduction of personality pathology in both groups (EG and CG). Moreover, a more fine-grained analysis showed how, at certain phases (t_3,7,9_) of treatment, the EG reported greater reduction in symptoms. A graphical analysis of Fig. 2 is potentially indicative of greater therapeutic gain in early phases (between the first and second month) for EG that was maintained later, even until the follow-up phase. This would be consistent with a borderline significance (*p* = 0.59) of the comparison between EG and CG for primary outcome (see Table 3).

**Fig. 4:**
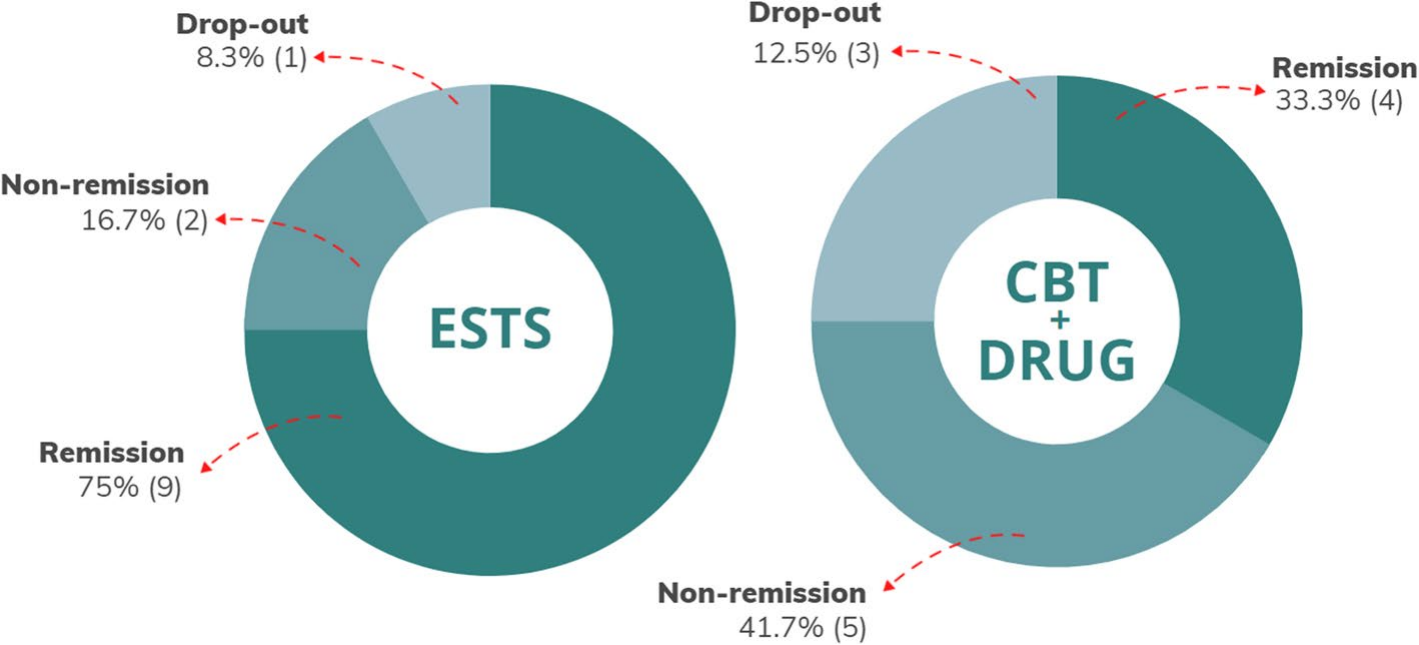
Rates of remission from diagnosis and drop-outs. *Note*. We report rates of remission from diagnosis of schizotypal personality disorder and drop-outs at 1-month follow-up. Percentages (raw numbers in brackets) are calculated in respect to the total number of randomized patients per arm (*n* = 12 + 12), that is those receiving Evolutionary Systems Therapy for Schizotypy (ESTS) in the experimental group and those receiving Cognitive Behavioral Therapy plus psychopharmacological treatment (CBT + Drug) in the control group, respectively

Similarly, two other measures included in the study as secondary outcomes (general symptomatology and metacognition) suggested a possible superiority of the experimental treatment, even if this is in contrast with the non-significance of the differences in remission from diagnosis (see Fig. 3). That said, remission rates in EG were more than double those in CG (EG = 9; CG = 4), and dropout rates were one-third (EG = 1; CG = 3). Figure 4 is indicative of the differences between the two arms in terms of drop-out, remission and non-remission.

These results may be consistent either with a variance caused by the low sample size and other biases or with a greater effectiveness of the experimental treatment. It is possible that a destigmatizing approach to conceptualization, based on an evolutionary model focused on the metacognitive functioning together with the use of experiential techniques aimed at increasing compassion and reactivating the capacity to soothe one’s own suffering may explain this result. Indeed, these two components are distinctive of the early phases of ESTS and potentially different with respect to a good psychiatric management (i.e. CBT and mandatory medication management). CBT’s focus on categorical diagnosis and the use of medication such as antipsychotics may have been perceived as more stigmatizing and so fueling the threat system of the clients. By assuming a possible vicious cycle between reduced ability to experience compassion and self-soothe, and impaired metacognition in those diagnosed with SPD [26, 44], we may hypothesize how an early intervention targeting these mechanisms would foster recovery. If a patient perceives elements of the therapeutic setting (e.g. categorical diagnosis; medication) as judgmental or coercive this would fuel a threatening response and reduce a nuanced understanding of subjective and intersubjective experience. Indeed, research suggests that internalized stigma in those presenting schizotypal features is associated with a reduced metacognitive functioning and increasingly threatening beliefs about self and others [60, 61]. This stigmatizing pattern may have been exacerbated by the mandatory psychopharmacological treatment.

The present study has several limitations, two of which must be carefully considered, including: A small sample size and possible therapist effect bias. First, the number of randomized patients did not allow for generalizing the results across the whole reference population of persons diagnosed with SPD. The registered sample size calculation was consistent with the pilot and exploratory nature of the RCT. We collected promising evidence (e.g. a larger increase of metacognition and larger reduction of general symptomatology in the EG) to confirm subsequently. Second, we opted for recruiting only two therapists, and this limited number may have resulted in a reduced quality of the study, such that reported outcomes may be more indicative of therapists’ competencies than interventions effectiveness. On the other hand, if we would have opted for recruiting more therapists, the variance in their competences and skills would have probably represented another significant bias and limitation of the study: EG is a novel treatment that requires diversified skills and few therapists have experience in treating SPD regardless of the approach used. A cost–benefit assessment prompted us to reduce a source of variance by defining tight inclusion criteria to choose the two therapists closest to an ideal training curriculum that does not exist yet. Future studies should confirm our preliminary results in a larger sample and through a more rigorous RCT. More specifically, it would be pivotal to recruit several therapists to randomize and allocate between two specific curricula in order to be trained in the two treatment conditions.

We would consider at least three other limitations. The primary outcome was assessed through a measure (i.e. PID-5-BF) that has several advantages for the patients, including its brevity and simplicity, however at the same time scholars have raised criticisms concerning its reliability [62]. Second, in our sample, patients showed a limited propensity to use psychiatric drugs. Given the limited sample size, this attitude could have negatively affected the CG where the medication was mandatory. In future studies, it may be desirable to consider treating SPD with CBT alone (i.e. without psychopharmacological medication) based on patient preference. Finally, a one-month follow-up is possibly a limited indicator of the stability of the changes achieved. By considering the severity of schizotypal symptoms and traits future studies should consider longer follow-ups. In conclusion, the generalizability of our results is primarily limited to the usefulness of a similar study in fostering future research on an understudied area of psychotherapy such as SPD.

## Conclusion

In this pilot RCT on repeated measures we tested a non-inferiority hypothesis in comparing a specifically designed therapy for SPD and a presumably good psychiatric management. The former—namely, ESTS—was a recovery-oriented therapy integrating metacognitively oriented, compassion focused and evolutionary approaches and, the latter was a combination of CBT for PDs and psychopharmacological treatment.

In spite of the low sample size and the preliminary nature of the study, results showed non-significant differences in primary outcome (i.e. personality pathology), whereas differences in secondary outcomes (i.e. remission from diagnosis, general symptomatology, and metacognition) possibly suggested a superiority of ESTS.

The main implication of this research is that an adequately powered effectiveness trial on large sample size is needed to provide evidence about relative effectiveness of ESTS and CBT plus psychopharmacological treatment for those diagnosed with SPD. A further implication is that ESTS can be considered eligible to be evaluated in terms of clinical efficacy and utility in treating those with schizotypal traits. We suggest considering SPD as a treatable condition, metacognition and social safeness as target mechanisms, and evolutionary psychopathology as theoretical framework for the intervention.

## Supplementary Information

**Additional file 1. **

## Data Availability

The data that support the findings of this study together with subsidiary analyses are openly available in Open Science Framework (OSF) at https://doi.org/10.17605/OSF.IO/BJ5ER
