# Supplementary material for: A pilot randomized controlled trial comparing a novel compassion and metacognition approach for schizotypal personality disorder with a combination of cognitive therapy and psychopharmacological treatment
Source: BMC Psychiatry. 2023 Feb 20;23:113. doi: 10.1186/s12888-023-04610-5 (PMC9942388; doi:10.1186/s12888-023-04610-5)
Supplement: Supplementary file 1 — Additional file 1. [file 12888_2023_4610_MOESM1_ESM.rtf]

SUPPLEMENTARY MATERIALS


In the following pages we report the complete outputs of the analyses presented in the paper plus subsidiary analyses. All the outputs were generated by SPSS version 25.

Crosstabs


Case Processing Summary	
	Cases	
	Valid	Missing	Total	
	N	Percent	N	Percent	N	Percent	
Sex * Harm	24	100,0%	0	0,0%	24	100,0%	


Sex * Harm Crosstabulation	
	Harm	Total	
	Experimental Group	Control Group		
Sex	Male	Count	7	8	15	
		% within Sex	46,7%	53,3%	100,0%	
		% within Harm	58,3%	66,7%	62,5%	
		% of Total	29,2%	33,3%	62,5%	
	Female	Count	5	4	9	
		% within Sex	55,6%	44,4%	100,0%	
		% within Harm	41,7%	33,3%	37,5%	
		% of Total	20,8%	16,7%	37,5%	
Total	Count	12	12	24	
	% within Sex	50,0%	50,0%	100,0%	
	% within Harm	100,0%	100,0%	100,0%	
	% of Total	50,0%	50,0%	100,0%	


Chi-Square Tests	
	Value	df	Asymptotic Significance (2-sided)	Exact Sig. (2-sided)	Exact Sig. (1-sided)	
Pearson Chi-Square	,178a	1	,673			
Continuity Correctionb	,000	1	1,000			
Likelihood Ratio	,178	1	,673			
Fisher's Exact Test				1,000	,500	
Linear-by-Linear Association	,170	1	,680			
N of Valid Cases	24					

a. 2 cells (50,0%) have expected count less than 5. The minimum expected count is 4,50.	
b. Computed only for a 2x2 table	


Risk Estimate	
	Value	95% Confidence Interval	
		Lower	Upper	
Odds Ratio for Sex (Male / Female)	,700	,133	3,684	
For cohort Harm = Experimental Group	,840	,379	1,863	
For cohort Harm = Control Group	1,200	,503	2,865	
N of Valid Cases	24			

ONEWAY Age BY Harm
  /MISSING ANALYSIS.


Oneway


ANOVA	
Age  	
	Sum of Squares	df	Mean Square	F	Sig.	
Between Groups	2,667	1	2,667	,233	,634	
Within Groups	251,833	22	11,447			
Total	254,500	23				

ONEWAY Education BY Harm
  /MISSING ANALYSIS.


Oneway

ANOVA	
Education  	
	Sum of Squares	df	Mean Square	F	Sig.	
Between Groups	,042	1	,042	,067	,797	
Within Groups	13,583	22	,617			
Total	13,625	23				

ONEWAY Income BY Harm
  /MISSING ANALYSIS.


Oneway

ANOVA	
Annual family income  	
	Sum of Squares	df	Mean Square	F	Sig.	
Between Groups	,000	1	,000	,000	1,000	
Within Groups	11,333	22	,515			
Total	11,333	23				


General Linear Model


Within-Subjects Factors	
Measure:   MEASURE_1  	
factor1	Dependent Variable	
1	GSIT2	
2	GSIT8	


Between-Subjects Factors	
	Value Label	N	
Harm	0	Experimental Group	11	
	1	Control Group	9	


Descriptive Statistics	
	Harm	Mean	Std. Deviation	N	
SCL90R Total score at T2	Experimental Group	113,09	8,396	11	
	Control Group	111,11	9,130	9	
	Total	112,20	8,557	20	
SCL90R Total score at T8	Experimental Group	45,18	8,072	11	
	Control Group	57,67	6,557	9	
	Total	50,80	9,644	20	


Multivariate Testsa	
Effect	Value	F	Hypothesis df	Error df	Sig.	
factor1	Pillai's Trace	,989	1601,877b	1,000	18,000	,000	
	Wilks' Lambda	,011	1601,877b	1,000	18,000	,000	
	Hotelling's Trace	88,993	1601,877b	1,000	18,000	,000	
	Roy's Largest Root	88,993	1601,877b	1,000	18,000	,000	
factor1 * Harm	Pillai's Trace	,558	22,758b	1,000	18,000	,000	
	Wilks' Lambda	,442	22,758b	1,000	18,000	,000	
	Hotelling's Trace	1,264	22,758b	1,000	18,000	,000	
	Roy's Largest Root	1,264	22,758b	1,000	18,000	,000	

Multivariate Testsa	
Effect	Partial Eta Squared	Noncent. Parameter	Observed Powerc	
factor1	Pillai's Trace	,989	1601,877	1,000	
	Wilks' Lambda	,989	1601,877	1,000	
	Hotelling's Trace	,989	1601,877	1,000	
	Roy's Largest Root	,989	1601,877	1,000	
factor1 * Harm	Pillai's Trace	,558	22,758	,995	
	Wilks' Lambda	,558	22,758	,995	
	Hotelling's Trace	,558	22,758	,995	
	Roy's Largest Root	,558	22,758	,995	

a. Design: Intercept + Harm 
 Within Subjects Design: factor1	
b. Exact statistic	
c. Computed using alpha = ,05	


Mauchly's Test of Sphericitya	
Measure:   MEASURE_1  	
Within Subjects Effect	Mauchly's W	Approx. Chi-Square	df	Sig.	Epsilonb	
					Greenhouse-Geisser	
factor1	1,000	,000	0	.	1,000	

Mauchly's Test of Sphericitya	
Measure:   MEASURE_1  	
Within Subjects Effect	Epsilon	
	Huynh-Feldt	Lower-bound	
factor1	1,000	1,000	

Tests the null hypothesis that the error covariance matrix of the orthonormalized transformed dependent variables is proportional to an identity matrix.a	
a. Design: Intercept + Harm 
 Within Subjects Design: factor1	
b. May be used to adjust the degrees of freedom for the averaged tests of significance. Corrected tests are displayed in the Tests of Within-Subjects Effects table.	


Tests of Within-Subjects Effects	
Measure:   MEASURE_1  	
Source	Type III Sum of Squares	df	Mean Square	F	
factor1	Sphericity Assumed	36448,534	1	36448,534	1601,877	
	Greenhouse-Geisser	36448,534	1,000	36448,534	1601,877	
	Huynh-Feldt	36448,534	1,000	36448,534	1601,877	
	Lower-bound	36448,534	1,000	36448,534	1601,877	
factor1 * Harm	Sphericity Assumed	517,834	1	517,834	22,758	
	Greenhouse-Geisser	517,834	1,000	517,834	22,758	
	Huynh-Feldt	517,834	1,000	517,834	22,758	
	Lower-bound	517,834	1,000	517,834	22,758	
Error(factor1)	Sphericity Assumed	409,566	18	22,754		
	Greenhouse-Geisser	409,566	18,000	22,754		
	Huynh-Feldt	409,566	18,000	22,754		
	Lower-bound	409,566	18,000	22,754		

Tests of Within-Subjects Effects	
Measure:   MEASURE_1  	
Source	Sig.	Partial Eta Squared	Noncent. Parameter	Observed Powera	
factor1	Sphericity Assumed	,000	,989	1601,877	1,000	
	Greenhouse-Geisser	,000	,989	1601,877	1,000	
	Huynh-Feldt	,000	,989	1601,877	1,000	
	Lower-bound	,000	,989	1601,877	1,000	
factor1 * Harm	Sphericity Assumed	,000	,558	22,758	,995	
	Greenhouse-Geisser	,000	,558	22,758	,995	
	Huynh-Feldt	,000	,558	22,758	,995	
	Lower-bound	,000	,558	22,758	,995	
Error(factor1)	Sphericity Assumed					
	Greenhouse-Geisser					
	Huynh-Feldt					
	Lower-bound					

a. Computed using alpha = ,05	


Tests of Within-Subjects Contrasts	
Measure:   MEASURE_1  	
Source	factor1	Type III Sum of Squares	df	Mean Square	F	Sig.	
factor1	Level 1 vs. Level 2	72897,069	1	72897,069	1601,877	,000	
factor1 * Harm	Level 1 vs. Level 2	1035,669	1	1035,669	22,758	,000	
Error(factor1)	Level 1 vs. Level 2	819,131	18	45,507			

Tests of Within-Subjects Contrasts	
Measure:   MEASURE_1  	
Source	factor1	Partial Eta Squared	Noncent. Parameter	Observed Powera	
factor1	Level 1 vs. Level 2	,989	1601,877	1,000	
factor1 * Harm	Level 1 vs. Level 2	,558	22,758	,995	
Error(factor1)	Level 1 vs. Level 2				

a. Computed using alpha = ,05	


Tests of Between-Subjects Effects	
Measure:   MEASURE_1  	
Transformed Variable:   Average  	
Source	Type III Sum of Squares	df	Mean Square	F	Sig.	Partial Eta Squared	
Intercept	132365,516	1	132365,516	2433,850	,000	,993	
Harm	136,566	1	136,566	2,511	,130	,122	
Error	978,934	18	54,385				

Tests of Between-Subjects Effects	
Measure:   MEASURE_1  	
Transformed Variable:   Average  	
Source	Noncent. Parameter	Observed Powera	
Intercept	2433,850	1,000	
Harm	2,511	,323	
Error			

a. Computed using alpha = ,05	


Custom Hypothesis Tests


Contrast Results (K Matrix)	
Harm Simple Contrasta	Averaged Variable	
	MEASURE_1	
Level 1 vs. Level 2	Contrast Estimate	-5,253	
	Hypothesized Value	0	
	Difference (Estimate - Hypothesized)	-5,253	
	Std. Error	3,315	
	Sig.	,130	
	95% Confidence Interval for Difference	Lower Bound	-12,216	
		Upper Bound	1,711	

a. Reference category = 2	


Test Results	
Measure:   MEASURE_1  	
Transformed Variable:   AVERAGE  	
Source	Sum of Squares	df	Mean Square	F	Sig.	Partial Eta Squared	
Contrast	136,566	1	136,566	2,511	,130	,122	
Error	978,934	18	54,385				

Test Results	
Measure:   MEASURE_1  	
Transformed Variable:   AVERAGE  	
Source	Noncent. Parameter	Observed Powera	
Contrast	2,511	,323	
Error			

a. Computed using alpha = ,05	

DATASET ACTIVATE DataSet1.

SAVE OUTFILE='C:\Users\dottc\Documents\SPD\SPD_RCT.sav'
  /COMPRESSED.
General Linear Model


Within-Subjects Factors	
Measure:   MEASURE_1  	
factor1	Dependent Variable	
1	MASAT2	
2	MASAT8	


Between-Subjects Factors	
	Value Label	N	
Harm	0	Experimental Group	11	
	1	Control Group	9	


Descriptive Statistics	
	Harm	Mean	Std. Deviation	N	
MAS-A Total score at T2	Experimental Group	10,36	2,618	11	
	Control Group	10,00	2,291	9	
	Total	10,20	2,419	20	
MAS-A Total score at T8	Experimental Group	22,82	1,601	11	
	Control Group	17,56	1,667	9	
	Total	20,45	3,120	20	


Multivariate Testsa	
Effect	Value	F	Hypothesis df	Error df	Sig.	
factor1	Pillai's Trace	,979	830,650b	1,000	18,000	,000	
	Wilks' Lambda	,021	830,650b	1,000	18,000	,000	
	Hotelling's Trace	46,147	830,650b	1,000	18,000	,000	
	Roy's Largest Root	46,147	830,650b	1,000	18,000	,000	
factor1 * Harm	Pillai's Trace	,734	49,789b	1,000	18,000	,000	
	Wilks' Lambda	,266	49,789b	1,000	18,000	,000	
	Hotelling's Trace	2,766	49,789b	1,000	18,000	,000	
	Roy's Largest Root	2,766	49,789b	1,000	18,000	,000	

Multivariate Testsa	
Effect	Partial Eta Squared	Noncent. Parameter	Observed Powerc	
factor1	Pillai's Trace	,979	830,650	1,000	
	Wilks' Lambda	,979	830,650	1,000	
	Hotelling's Trace	,979	830,650	1,000	
	Roy's Largest Root	,979	830,650	1,000	
factor1 * Harm	Pillai's Trace	,734	49,789	1,000	
	Wilks' Lambda	,734	49,789	1,000	
	Hotelling's Trace	,734	49,789	1,000	
	Roy's Largest Root	,734	49,789	1,000	

a. Design: Intercept + Harm 
 Within Subjects Design: factor1	
b. Exact statistic	
c. Computed using alpha = ,05	


Mauchly's Test of Sphericitya	
Measure:   MEASURE_1  	
Within Subjects Effect	Mauchly's W	Approx. Chi-Square	df	Sig.	Epsilonb	
					Greenhouse-Geisser	
factor1	1,000	,000	0	.	1,000	

Mauchly's Test of Sphericitya	
Measure:   MEASURE_1  	
Within Subjects Effect	Epsilon	
	Huynh-Feldt	Lower-bound	
factor1	1,000	1,000	

Tests the null hypothesis that the error covariance matrix of the orthonormalized transformed dependent variables is proportional to an identity matrix.a	
a. Design: Intercept + Harm 
 Within Subjects Design: factor1	
b. May be used to adjust the degrees of freedom for the averaged tests of significance. Corrected tests are displayed in the Tests of Within-Subjects Effects table.	


Tests of Within-Subjects Effects	
Measure:   MEASURE_1  	
Source	Type III Sum of Squares	df	Mean Square	F	
factor1	Sphericity Assumed	991,000	1	991,000	830,650	
	Greenhouse-Geisser	991,000	1,000	991,000	830,650	
	Huynh-Feldt	991,000	1,000	991,000	830,650	
	Lower-bound	991,000	1,000	991,000	830,650	
factor1 * Harm	Sphericity Assumed	59,400	1	59,400	49,789	
	Greenhouse-Geisser	59,400	1,000	59,400	49,789	
	Huynh-Feldt	59,400	1,000	59,400	49,789	
	Lower-bound	59,400	1,000	59,400	49,789	
Error(factor1)	Sphericity Assumed	21,475	18	1,193		
	Greenhouse-Geisser	21,475	18,000	1,193		
	Huynh-Feldt	21,475	18,000	1,193		
	Lower-bound	21,475	18,000	1,193		

Tests of Within-Subjects Effects	
Measure:   MEASURE_1  	
Source	Sig.	Partial Eta Squared	Noncent. Parameter	Observed Powera	
factor1	Sphericity Assumed	,000	,979	830,650	1,000	
	Greenhouse-Geisser	,000	,979	830,650	1,000	
	Huynh-Feldt	,000	,979	830,650	1,000	
	Lower-bound	,000	,979	830,650	1,000	
factor1 * Harm	Sphericity Assumed	,000	,734	49,789	1,000	
	Greenhouse-Geisser	,000	,734	49,789	1,000	
	Huynh-Feldt	,000	,734	49,789	1,000	
	Lower-bound	,000	,734	49,789	1,000	
Error(factor1)	Sphericity Assumed					
	Greenhouse-Geisser					
	Huynh-Feldt					
	Lower-bound					

a. Computed using alpha = ,05	


Tests of Within-Subjects Contrasts	
Measure:   MEASURE_1  	
Source	factor1	Type III Sum of Squares	df	Mean Square	F	Sig.	
factor1	Level 1 vs. Level 2	1982,001	1	1982,001	830,650	,000	
factor1 * Harm	Level 1 vs. Level 2	118,801	1	118,801	49,789	,000	
Error(factor1)	Level 1 vs. Level 2	42,949	18	2,386			

Tests of Within-Subjects Contrasts	
Measure:   MEASURE_1  	
Source	factor1	Partial Eta Squared	Noncent. Parameter	Observed Powera	
factor1	Level 1 vs. Level 2	,979	830,650	1,000	
factor1 * Harm	Level 1 vs. Level 2	,734	49,789	1,000	
Error(factor1)	Level 1 vs. Level 2				

a. Computed using alpha = ,05	


Tests of Between-Subjects Effects	
Measure:   MEASURE_1  	
Transformed Variable:   Average  	
Source	Type III Sum of Squares	df	Mean Square	F	Sig.	Partial Eta Squared	
Intercept	4565,173	1	4565,173	1200,227	,000	,985	
Harm	39,173	1	39,173	10,299	,005	,364	
Error	68,465	18	3,804				

Tests of Between-Subjects Effects	
Measure:   MEASURE_1  	
Transformed Variable:   Average  	
Source	Noncent. Parameter	Observed Powera	
Intercept	1200,227	1,000	
Harm	10,299	,859	
Error			

a. Computed using alpha = ,05	


Custom Hypothesis Tests


Contrast Results (K Matrix)	
Harm Simple Contrasta	Averaged Variable	
	MEASURE_1	
Level 1 vs. Level 2	Contrast Estimate	2,813	
	Hypothesized Value	0	
	Difference (Estimate - Hypothesized)	2,813	
	Std. Error	,877	
	Sig.	,005	
	95% Confidence Interval for Difference	Lower Bound	,971	
		Upper Bound	4,655	

a. Reference category = 2	


Test Results	
Measure:   MEASURE_1  	
Transformed Variable:   AVERAGE  	
Source	Sum of Squares	df	Mean Square	F	Sig.	Partial Eta Squared	
Contrast	39,173	1	39,173	10,299	,005	,364	
Error	68,465	18	3,804				

Test Results	
Measure:   MEASURE_1  	
Transformed Variable:   AVERAGE  	
Source	Noncent. Parameter	Observed Powera	
Contrast	10,299	,859	
Error			

a. Computed using alpha = ,05	


General Linear Model


Within-Subjects Factors	
Measure:   MEASURE_1  	
factor1	Dependent Variable	
1	T1	
2	T2	
3	T3	
4	T4	
5	T5	
6	T6	
7	T7	
8	T8	
9	T9	


Between-Subjects Factors	
	Value Label	N	
Harm	0	Experimental Group	11	
	1	Control Group	9	


Multivariate Testsa	
Effect	Value	F	Hypothesis df	Error df	Sig.	
factor1	Pillai's Trace	,928	17,646b	8,000	11,000	,000	
	Wilks' Lambda	,072	17,646b	8,000	11,000	,000	
	Hotelling's Trace	12,834	17,646b	8,000	11,000	,000	
	Roy's Largest Root	12,834	17,646b	8,000	11,000	,000	
factor1 * Harm	Pillai's Trace	,766	4,491b	8,000	11,000	,012	
	Wilks' Lambda	,234	4,491b	8,000	11,000	,012	
	Hotelling's Trace	3,267	4,491b	8,000	11,000	,012	
	Roy's Largest Root	3,267	4,491b	8,000	11,000	,012	

Multivariate Testsa	
Effect	Partial Eta Squared	
factor1	Pillai's Trace	,928	
	Wilks' Lambda	,928	
	Hotelling's Trace	,928	
	Roy's Largest Root	,928	
factor1 * Harm	Pillai's Trace	,766	
	Wilks' Lambda	,766	
	Hotelling's Trace	,766	
	Roy's Largest Root	,766	

a. Design: Intercept + Harm 
 Within Subjects Design: factor1	
b. Exact statistic	


Mauchly's Test of Sphericitya	
Measure:   MEASURE_1  	
Within Subjects Effect	Mauchly's W	Approx. Chi-Square	df	Sig.	Epsilonb	
					Greenhouse-Geisser	
factor1	,001	106,839	35	,000	,343	

Mauchly's Test of Sphericitya	
Measure:   MEASURE_1  	
Within Subjects Effect	Epsilon	
	Huynh-Feldt	Lower-bound	
factor1	,433	,125	

Tests the null hypothesis that the error covariance matrix of the orthonormalized transformed dependent variables is proportional to an identity matrix.a	
a. Design: Intercept + Harm 
 Within Subjects Design: factor1	
b. May be used to adjust the degrees of freedom for the averaged tests of significance. Corrected tests are displayed in the Tests of Within-Subjects Effects table.	


Tests of Within-Subjects Effects	
Measure:   MEASURE_1  	
Source	Type III Sum of Squares	df	Mean Square	F	
factor1	Sphericity Assumed	15342,749	8	1917,844	92,277	
	Greenhouse-Geisser	15342,749	2,744	5592,312	92,277	
	Huynh-Feldt	15342,749	3,465	4427,315	92,277	
	Lower-bound	15342,749	1,000	15342,749	92,277	
factor1 * Harm	Sphericity Assumed	321,683	8	40,210	1,935	
	Greenhouse-Geisser	321,683	2,744	117,251	1,935	
	Huynh-Feldt	321,683	3,465	92,825	1,935	
	Lower-bound	321,683	1,000	321,683	1,935	
Error(factor1)	Sphericity Assumed	2992,828	144	20,784		
	Greenhouse-Geisser	2992,828	49,384	60,603		
	Huynh-Feldt	2992,828	62,379	47,978		
	Lower-bound	2992,828	18,000	166,268		

Tests of Within-Subjects Effects	
Measure:   MEASURE_1  	
Source	Sig.	Partial Eta Squared	
factor1	Sphericity Assumed	,000	,837	
	Greenhouse-Geisser	,000	,837	
	Huynh-Feldt	,000	,837	
	Lower-bound	,000	,837	
factor1 * Harm	Sphericity Assumed	,059	,097	
	Greenhouse-Geisser	,141	,097	
	Huynh-Feldt	,125	,097	
	Lower-bound	,181	,097	
Error(factor1)	Sphericity Assumed			
	Greenhouse-Geisser			
	Huynh-Feldt			
	Lower-bound			


Tests of Within-Subjects Contrasts	
Measure:   MEASURE_1  	
Source	factor1	Type III Sum of Squares	df	Mean Square	F	Sig.	
factor1	Level 2 vs. Level 1	,001	1	,001	,000	,995	
	Level 3 vs. Level 1	130,335	1	130,335	7,808	,012	
	Level 4 vs. Level 1	771,564	1	771,564	17,768	,001	
	Level 5 vs. Level 1	2930,950	1	2930,950	61,061	,000	
	Level 6 vs. Level 1	3554,752	1	3554,752	92,492	,000	
	Level 7 vs. Level 1	7174,425	1	7174,425	124,589	,000	
	Level 8 vs. Level 1	12440,085	1	12440,085	173,520	,000	
	Level 9 vs. Level 1	11481,729	1	11481,729	188,729	,000	
factor1 * Harm	Level 2 vs. Level 1	4,001	1	4,001	,369	,551	
	Level 3 vs. Level 1	108,735	1	108,735	6,514	,020	
	Level 4 vs. Level 1	131,364	1	131,364	3,025	,099	
	Level 5 vs. Level 1	158,950	1	158,950	3,311	,085	
	Level 6 vs. Level 1	124,752	1	124,752	3,246	,088	
	Level 7 vs. Level 1	340,425	1	340,425	5,912	,026	
	Level 8 vs. Level 1	160,085	1	160,085	2,233	,152	
	Level 9 vs. Level 1	347,929	1	347,929	5,719	,028	
Error(factor1)	Level 2 vs. Level 1	194,949	18	10,831			
	Level 3 vs. Level 1	300,465	18	16,692			
	Level 4 vs. Level 1	781,636	18	43,424			
	Level 5 vs. Level 1	864,000	18	48,000			
	Level 6 vs. Level 1	691,798	18	38,433			
	Level 7 vs. Level 1	1036,525	18	57,585			
	Level 8 vs. Level 1	1290,465	18	71,692			
	Level 9 vs. Level 1	1095,071	18	60,837			

Tests of Within-Subjects Contrasts	
Measure:   MEASURE_1  	
Source	factor1	Partial Eta Squared	
factor1	Level 2 vs. Level 1	,000	
	Level 3 vs. Level 1	,303	
	Level 4 vs. Level 1	,497	
	Level 5 vs. Level 1	,772	
	Level 6 vs. Level 1	,837	
	Level 7 vs. Level 1	,874	
	Level 8 vs. Level 1	,906	
	Level 9 vs. Level 1	,913	
factor1 * Harm	Level 2 vs. Level 1	,020	
	Level 3 vs. Level 1	,266	
	Level 4 vs. Level 1	,144	
	Level 5 vs. Level 1	,155	
	Level 6 vs. Level 1	,153	
	Level 7 vs. Level 1	,247	
	Level 8 vs. Level 1	,110	
	Level 9 vs. Level 1	,241	
Error(factor1)	Level 2 vs. Level 1		
	Level 3 vs. Level 1		
	Level 4 vs. Level 1		
	Level 5 vs. Level 1		
	Level 6 vs. Level 1		
	Level 7 vs. Level 1		
	Level 8 vs. Level 1		
	Level 9 vs. Level 1		


Tests of Between-Subjects Effects	
Measure:   MEASURE_1  	
Transformed Variable:   Average  	
Source	Type III Sum of Squares	df	Mean Square	F	Sig.	Partial Eta Squared	
Intercept	32978,371	1	32978,371	1041,689	,000	,983	
Harm	79,467	1	79,467	2,510	,131	,122	
Error	569,854	18	31,659				


Custom Hypothesis Tests


Contrast Results (K Matrix)	
Harm Simple Contrasta	Averaged Variable	
	MEASURE_1	
Level 2 vs. Level 1	Contrast Estimate	4,007	
	Hypothesized Value	0	
	Difference (Estimate - Hypothesized)	4,007	
	Std. Error	2,529	
	Sig.	,131	
	95% Confidence Interval for Difference	Lower Bound	-1,306	
		Upper Bound	9,320	

a. Reference category = 1	


Test Results	
Measure:   MEASURE_1  	
Transformed Variable:   AVERAGE  	
Source	Sum of Squares	df	Mean Square	F	Sig.	Partial Eta Squared	
Contrast	79,467	1	79,467	2,510	,131	,122	
Error	569,854	18	31,659				


Estimated Marginal Means


Harm * factor1	
Measure:   MEASURE_1  	
Harm	factor1	Mean	Std. Error	95% Confidence Interval	
				Lower Bound	Upper Bound	
Experimental Group	1	52,636	1,977	48,482	56,790	
	2	52,182	2,300	47,350	57,013	
	3	47,727	1,902	43,731	51,724	
	4	43,818	2,237	39,119	48,517	
	5	37,636	1,748	33,965	41,308	
	6	36,727	1,911	32,712	40,742	
	7	29,455	2,296	24,631	34,278	
	8	24,727	2,345	19,802	29,653	
	9	24,364	2,393	19,337	29,390	
Control Group	1	51,778	2,186	47,185	56,370	
	2	52,222	2,542	46,881	57,564	
	3	51,556	2,103	47,137	55,974	
	4	48,111	2,473	42,916	53,306	
	5	42,444	1,932	38,385	46,504	
	6	40,889	2,113	36,450	45,327	
	7	36,889	2,538	31,556	42,221	
	8	29,556	2,592	24,110	35,001	
	9	31,889	2,645	26,332	37,446	

GLM T1 T2 T3 T4 T5 T6 T7 T8 T9 BY Harm
  /WSFACTOR=factor1 9 Simple(1)
  /CONTRAST(Harm)=Simple(1)
  /METHOD=SSTYPE(3)
  /EMMEANS=TABLES(Harm*factor1)
  /PRINT=DESCRIPTIVE ETASQ
  /CRITERIA=ALPHA(.05)
  /WSDESIGN=factor1
  /DESIGN=Harm.


General Linear Model


Within-Subjects Factors	
Measure:   MEASURE_1  	
factor1	Dependent Variable	
1	T1	
2	T2	
3	T3	
4	T4	
5	T5	
6	T6	
7	T7	
8	T8	
9	T9	


Between-Subjects Factors	
	Value Label	N	
Harm	0	Experimental Group	11	
	1	Control Group	9	


Descriptive Statistics	
	Harm	Mean	Std. Deviation	N	
Pre-assessment	Experimental Group	52,64	8,286	11	
	Control Group	51,78	3,308	9	
	Total	52,25	6,398	20	
Initial assessment	Experimental Group	52,18	9,304	11	
	Control Group	52,22	4,764	9	
	Total	52,20	7,424	20	
1st month	Experimental Group	47,73	7,115	11	
	Control Group	51,56	5,126	9	
	Total	49,45	6,444	20	
2nd month	Experimental Group	43,82	7,441	11	
	Control Group	48,11	7,390	9	
	Total	45,75	7,545	20	
3rd month	Experimental Group	37,64	4,884	11	
	Control Group	42,44	6,766	9	
	Total	39,80	6,152	20	
4th month	Experimental Group	36,73	6,117	11	
	Control Group	40,89	6,604	9	
	Total	38,60	6,524	20	
5th month	Experimental Group	29,45	7,461	11	
	Control Group	36,89	7,801	9	
	Total	32,80	8,326	20	
Final assessment	Experimental Group	24,73	7,485	11	
	Control Group	29,56	8,126	9	
	Total	26,90	7,960	20	
Followup	Experimental Group	24,36	8,250	11	
	Control Group	31,89	7,524	9	
	Total	27,75	8,626	20	


Multivariate Testsa	
Effect	Value	F	Hypothesis df	Error df	Sig.	
factor1	Pillai's Trace	,928	17,646b	8,000	11,000	,000	
	Wilks' Lambda	,072	17,646b	8,000	11,000	,000	
	Hotelling's Trace	12,834	17,646b	8,000	11,000	,000	
	Roy's Largest Root	12,834	17,646b	8,000	11,000	,000	
factor1 * Harm	Pillai's Trace	,766	4,491b	8,000	11,000	,012	
	Wilks' Lambda	,234	4,491b	8,000	11,000	,012	
	Hotelling's Trace	3,267	4,491b	8,000	11,000	,012	
	Roy's Largest Root	3,267	4,491b	8,000	11,000	,012	

Multivariate Testsa	
Effect	Partial Eta Squared	
factor1	Pillai's Trace	,928	
	Wilks' Lambda	,928	
	Hotelling's Trace	,928	
	Roy's Largest Root	,928	
factor1 * Harm	Pillai's Trace	,766	
	Wilks' Lambda	,766	
	Hotelling's Trace	,766	
	Roy's Largest Root	,766	

a. Design: Intercept + Harm 
 Within Subjects Design: factor1	
b. Exact statistic	


Mauchly's Test of Sphericitya	
Measure:   MEASURE_1  	
Within Subjects Effect	Mauchly's W	Approx. Chi-Square	df	Sig.	Epsilonb	
					Greenhouse-Geisser	
factor1	,001	106,839	35	,000	,343	

Mauchly's Test of Sphericitya	
Measure:   MEASURE_1  	
Within Subjects Effect	Epsilon	
	Huynh-Feldt	Lower-bound	
factor1	,433	,125	

Tests the null hypothesis that the error covariance matrix of the orthonormalized transformed dependent variables is proportional to an identity matrix.a	
a. Design: Intercept + Harm 
 Within Subjects Design: factor1	
b. May be used to adjust the degrees of freedom for the averaged tests of significance. Corrected tests are displayed in the Tests of Within-Subjects Effects table.	


Tests of Within-Subjects Effects	
Measure:   MEASURE_1  	
Source	Type III Sum of Squares	df	Mean Square	F	
factor1	Sphericity Assumed	15342,749	8	1917,844	92,277	
	Greenhouse-Geisser	15342,749	2,744	5592,312	92,277	
	Huynh-Feldt	15342,749	3,465	4427,315	92,277	
	Lower-bound	15342,749	1,000	15342,749	92,277	
factor1 * Harm	Sphericity Assumed	321,683	8	40,210	1,935	
	Greenhouse-Geisser	321,683	2,744	117,251	1,935	
	Huynh-Feldt	321,683	3,465	92,825	1,935	
	Lower-bound	321,683	1,000	321,683	1,935	
Error(factor1)	Sphericity Assumed	2992,828	144	20,784		
	Greenhouse-Geisser	2992,828	49,384	60,603		
	Huynh-Feldt	2992,828	62,379	47,978		
	Lower-bound	2992,828	18,000	166,268		

Tests of Within-Subjects Effects	
Measure:   MEASURE_1  	
Source	Sig.	Partial Eta Squared	
factor1	Sphericity Assumed	,000	,837	
	Greenhouse-Geisser	,000	,837	
	Huynh-Feldt	,000	,837	
	Lower-bound	,000	,837	
factor1 * Harm	Sphericity Assumed	,059	,097	
	Greenhouse-Geisser	,141	,097	
	Huynh-Feldt	,125	,097	
	Lower-bound	,181	,097	
Error(factor1)	Sphericity Assumed			
	Greenhouse-Geisser			
	Huynh-Feldt			
	Lower-bound			


Tests of Within-Subjects Contrasts	
Measure:   MEASURE_1  	
Source	factor1	Type III Sum of Squares	df	Mean Square	F	Sig.	
factor1	Level 2 vs. Level 1	,001	1	,001	,000	,995	
	Level 3 vs. Level 1	130,335	1	130,335	7,808	,012	
	Level 4 vs. Level 1	771,564	1	771,564	17,768	,001	
	Level 5 vs. Level 1	2930,950	1	2930,950	61,061	,000	
	Level 6 vs. Level 1	3554,752	1	3554,752	92,492	,000	
	Level 7 vs. Level 1	7174,425	1	7174,425	124,589	,000	
	Level 8 vs. Level 1	12440,085	1	12440,085	173,520	,000	
	Level 9 vs. Level 1	11481,729	1	11481,729	188,729	,000	
factor1 * Harm	Level 2 vs. Level 1	4,001	1	4,001	,369	,551	
	Level 3 vs. Level 1	108,735	1	108,735	6,514	,020	
	Level 4 vs. Level 1	131,364	1	131,364	3,025	,099	
	Level 5 vs. Level 1	158,950	1	158,950	3,311	,085	
	Level 6 vs. Level 1	124,752	1	124,752	3,246	,088	
	Level 7 vs. Level 1	340,425	1	340,425	5,912	,026	
	Level 8 vs. Level 1	160,085	1	160,085	2,233	,152	
	Level 9 vs. Level 1	347,929	1	347,929	5,719	,028	
Error(factor1)	Level 2 vs. Level 1	194,949	18	10,831			
	Level 3 vs. Level 1	300,465	18	16,692			
	Level 4 vs. Level 1	781,636	18	43,424			
	Level 5 vs. Level 1	864,000	18	48,000			
	Level 6 vs. Level 1	691,798	18	38,433			
	Level 7 vs. Level 1	1036,525	18	57,585			
	Level 8 vs. Level 1	1290,465	18	71,692			
	Level 9 vs. Level 1	1095,071	18	60,837			

Tests of Within-Subjects Contrasts	
Measure:   MEASURE_1  	
Source	factor1	Partial Eta Squared	
factor1	Level 2 vs. Level 1	,000	
	Level 3 vs. Level 1	,303	
	Level 4 vs. Level 1	,497	
	Level 5 vs. Level 1	,772	
	Level 6 vs. Level 1	,837	
	Level 7 vs. Level 1	,874	
	Level 8 vs. Level 1	,906	
	Level 9 vs. Level 1	,913	
factor1 * Harm	Level 2 vs. Level 1	,020	
	Level 3 vs. Level 1	,266	
	Level 4 vs. Level 1	,144	
	Level 5 vs. Level 1	,155	
	Level 6 vs. Level 1	,153	
	Level 7 vs. Level 1	,247	
	Level 8 vs. Level 1	,110	
	Level 9 vs. Level 1	,241	
Error(factor1)	Level 2 vs. Level 1		
	Level 3 vs. Level 1		
	Level 4 vs. Level 1		
	Level 5 vs. Level 1		
	Level 6 vs. Level 1		
	Level 7 vs. Level 1		
	Level 8 vs. Level 1		
	Level 9 vs. Level 1		


Tests of Between-Subjects Effects	
Measure:   MEASURE_1  	
Transformed Variable:   Average  	
Source	Type III Sum of Squares	df	Mean Square	F	Sig.	Partial Eta Squared	
Intercept	32978,371	1	32978,371	1041,689	,000	,983	
Harm	79,467	1	79,467	2,510	,131	,122	
Error	569,854	18	31,659				


Custom Hypothesis Tests


Contrast Results (K Matrix)	
Harm Simple Contrasta	Averaged Variable	
	MEASURE_1	
Level 2 vs. Level 1	Contrast Estimate	4,007	
	Hypothesized Value	0	
	Difference (Estimate - Hypothesized)	4,007	
	Std. Error	2,529	
	Sig.	,131	
	95% Confidence Interval for Difference	Lower Bound	-1,306	
		Upper Bound	9,320	

a. Reference category = 1	


Test Results	
Measure:   MEASURE_1  	
Transformed Variable:   AVERAGE  	
Source	Sum of Squares	df	Mean Square	F	Sig.	Partial Eta Squared	
Contrast	79,467	1	79,467	2,510	,131	,122	
Error	569,854	18	31,659				


Estimated Marginal Means


Harm * factor1	
Measure:   MEASURE_1  	
Harm	factor1	Mean	Std. Error	95% Confidence Interval	
				Lower Bound	Upper Bound	
Experimental Group	1	52,636	1,977	48,482	56,790	
	2	52,182	2,300	47,350	57,013	
	3	47,727	1,902	43,731	51,724	
	4	43,818	2,237	39,119	48,517	
	5	37,636	1,748	33,965	41,308	
	6	36,727	1,911	32,712	40,742	
	7	29,455	2,296	24,631	34,278	
	8	24,727	2,345	19,802	29,653	
	9	24,364	2,393	19,337	29,390	
Control Group	1	51,778	2,186	47,185	56,370	
	2	52,222	2,542	46,881	57,564	
	3	51,556	2,103	47,137	55,974	
	4	48,111	2,473	42,916	53,306	
	5	42,444	1,932	38,385	46,504	
	6	40,889	2,113	36,450	45,327	
	7	36,889	2,538	31,556	42,221	
	8	29,556	2,592	24,110	35,001	
	9	31,889	2,645	26,332	37,446	

GLM GSIT2 GSIT8 BY Harm
  /WSFACTOR=Factor 2 Simple(1)
  /CONTRAST(Harm)=Simple(1)
  /METHOD=SSTYPE(3)
  /PRINT=DESCRIPTIVE ETASQ
  /CRITERIA=ALPHA(.05)
  /WSDESIGN=Factor
  /DESIGN=Harm.


General Linear Model

Within-Subjects Factors	
Measure:   MEASURE_1  	
Factor	Dependent Variable	
1	GSIT2	
2	GSIT8	


Between-Subjects Factors	
	Value Label	N	
Harm	0	Experimental Group	11	
	1	Control Group	9	


Descriptive Statistics	
	Harm	Mean	Std. Deviation	N	
SCL90R Total score at T2	Experimental Group	113,09	8,396	11	
	Control Group	111,11	9,130	9	
	Total	112,20	8,557	20	
SCL90R Total score at T9	Experimental Group	45,18	8,072	11	
	Control Group	57,67	6,557	9	
	Total	50,80	9,644	20	


Multivariate Testsa	
Effect	Value	F	Hypothesis df	Error df	Sig.	
Factor	Pillai's Trace	,989	1601,877b	1,000	18,000	,000	
	Wilks' Lambda	,011	1601,877b	1,000	18,000	,000	
	Hotelling's Trace	88,993	1601,877b	1,000	18,000	,000	
	Roy's Largest Root	88,993	1601,877b	1,000	18,000	,000	
Factor * Harm	Pillai's Trace	,558	22,758b	1,000	18,000	,000	
	Wilks' Lambda	,442	22,758b	1,000	18,000	,000	
	Hotelling's Trace	1,264	22,758b	1,000	18,000	,000	
	Roy's Largest Root	1,264	22,758b	1,000	18,000	,000	

Multivariate Testsa	
Effect	Partial Eta Squared	
Factor	Pillai's Trace	,989	
	Wilks' Lambda	,989	
	Hotelling's Trace	,989	
	Roy's Largest Root	,989	
Factor * Harm	Pillai's Trace	,558	
	Wilks' Lambda	,558	
	Hotelling's Trace	,558	
	Roy's Largest Root	,558	

a. Design: Intercept + Harm 
 Within Subjects Design: Factor	
b. Exact statistic	


Mauchly's Test of Sphericitya	
Measure:   MEASURE_1  	
Within Subjects Effect	Mauchly's W	Approx. Chi-Square	df	Sig.	Epsilonb	
					Greenhouse-Geisser	
Factor	1,000	,000	0	.	1,000	

Mauchly's Test of Sphericitya	
Measure:   MEASURE_1  	
Within Subjects Effect	Epsilon	
	Huynh-Feldt	Lower-bound	
Factor	1,000	1,000	

Tests the null hypothesis that the error covariance matrix of the orthonormalized transformed dependent variables is proportional to an identity matrix.a	
a. Design: Intercept + Harm 
 Within Subjects Design: Factor	
b. May be used to adjust the degrees of freedom for the averaged tests of significance. Corrected tests are displayed in the Tests of Within-Subjects Effects table.	


Tests of Within-Subjects Effects	
Measure:   MEASURE_1  	
Source	Type III Sum of Squares	df	Mean Square	F	
Factor	Sphericity Assumed	36448,534	1	36448,534	1601,877	
	Greenhouse-Geisser	36448,534	1,000	36448,534	1601,877	
	Huynh-Feldt	36448,534	1,000	36448,534	1601,877	
	Lower-bound	36448,534	1,000	36448,534	1601,877	
Factor * Harm	Sphericity Assumed	517,834	1	517,834	22,758	
	Greenhouse-Geisser	517,834	1,000	517,834	22,758	
	Huynh-Feldt	517,834	1,000	517,834	22,758	
	Lower-bound	517,834	1,000	517,834	22,758	
Error(Factor)	Sphericity Assumed	409,566	18	22,754		
	Greenhouse-Geisser	409,566	18,000	22,754		
	Huynh-Feldt	409,566	18,000	22,754		
	Lower-bound	409,566	18,000	22,754		

Tests of Within-Subjects Effects	
Measure:   MEASURE_1  	
Source	Sig.	Partial Eta Squared	
Factor	Sphericity Assumed	,000	,989	
	Greenhouse-Geisser	,000	,989	
	Huynh-Feldt	,000	,989	
	Lower-bound	,000	,989	
Factor * Harm	Sphericity Assumed	,000	,558	
	Greenhouse-Geisser	,000	,558	
	Huynh-Feldt	,000	,558	
	Lower-bound	,000	,558	
Error(Factor)	Sphericity Assumed			
	Greenhouse-Geisser			
	Huynh-Feldt			
	Lower-bound			


Tests of Within-Subjects Contrasts	
Measure:   MEASURE_1  	
Source	Factor	Type III Sum of Squares	df	Mean Square	F	Sig.	
Factor	Level 2 vs. Level 1	72897,069	1	72897,069	1601,877	,000	
Factor * Harm	Level 2 vs. Level 1	1035,669	1	1035,669	22,758	,000	
Error(Factor)	Level 2 vs. Level 1	819,131	18	45,507			

Tests of Within-Subjects Contrasts	
Measure:   MEASURE_1  	
Source	Factor	Partial Eta Squared	
Factor	Level 2 vs. Level 1	,989	
Factor * Harm	Level 2 vs. Level 1	,558	
Error(Factor)	Level 2 vs. Level 1		


Tests of Between-Subjects Effects	
Measure:   MEASURE_1  	
Transformed Variable:   Average  	
Source	Type III Sum of Squares	df	Mean Square	F	Sig.	Partial Eta Squared	
Intercept	132365,516	1	132365,516	2433,850	,000	,993	
Harm	136,566	1	136,566	2,511	,130	,122	
Error	978,934	18	54,385				


Custom Hypothesis Tests


Contrast Results (K Matrix)	
Harm Simple Contrasta	Averaged Variable	
	MEASURE_1	
Level 2 vs. Level 1	Contrast Estimate	5,253	
	Hypothesized Value	0	
	Difference (Estimate - Hypothesized)	5,253	
	Std. Error	3,315	
	Sig.	,130	
	95% Confidence Interval for Difference	Lower Bound	-1,711	
		Upper Bound	12,216	

a. Reference category = 1	


Test Results	
Measure:   MEASURE_1  	
Transformed Variable:   AVERAGE  	
Source	Sum of Squares	df	Mean Square	F	Sig.	Partial Eta Squared	
Contrast	136,566	1	136,566	2,511	,130	,122	
Error	978,934	18	54,385				

GLM MASAT2 MASAT8 BY Harm
  /WSFACTOR=Factor 2 Simple(1)
  /CONTRAST(Harm)=Simple(1)
  /METHOD=SSTYPE(3)
  /PRINT=DESCRIPTIVE ETASQ
  /CRITERIA=ALPHA(.05)
  /WSDESIGN=Factor
  /DESIGN=Harm.


General Linear Model


Within-Subjects Factors	
Measure:   MEASURE_1  	
Factor	Dependent Variable	
1	MASAT2	
2	MASAT8	


Between-Subjects Factors	
	Value Label	N	
Harm	0	Experimental Group	11	
	1	Control Group	9	


Descriptive Statistics	
	Harm	Mean	Std. Deviation	N	
MAS-A Total score at T2	Experimental Group	10,36	2,618	11	
	Control Group	10,00	2,291	9	
	Total	10,20	2,419	20	
MAS-A Total score at T9	Experimental Group	22,82	1,601	11	
	Control Group	17,56	1,667	9	
	Total	20,45	3,120	20	


Multivariate Testsa	
Effect	Value	F	Hypothesis df	Error df	Sig.	
Factor	Pillai's Trace	,979	830,650b	1,000	18,000	,000	
	Wilks' Lambda	,021	830,650b	1,000	18,000	,000	
	Hotelling's Trace	46,147	830,650b	1,000	18,000	,000	
	Roy's Largest Root	46,147	830,650b	1,000	18,000	,000	
Factor * Harm	Pillai's Trace	,734	49,789b	1,000	18,000	,000	
	Wilks' Lambda	,266	49,789b	1,000	18,000	,000	
	Hotelling's Trace	2,766	49,789b	1,000	18,000	,000	
	Roy's Largest Root	2,766	49,789b	1,000	18,000	,000	

Multivariate Testsa	
Effect	Partial Eta Squared	
Factor	Pillai's Trace	,979	
	Wilks' Lambda	,979	
	Hotelling's Trace	,979	
	Roy's Largest Root	,979	
Factor * Harm	Pillai's Trace	,734	
	Wilks' Lambda	,734	
	Hotelling's Trace	,734	
	Roy's Largest Root	,734	

a. Design: Intercept + Harm 
 Within Subjects Design: Factor	
b. Exact statistic	


Mauchly's Test of Sphericitya	
Measure:   MEASURE_1  	
Within Subjects Effect	Mauchly's W	Approx. Chi-Square	df	Sig.	Epsilonb	
					Greenhouse-Geisser	
Factor	1,000	,000	0	.	1,000	

Mauchly's Test of Sphericitya	
Measure:   MEASURE_1  	
Within Subjects Effect	Epsilon	
	Huynh-Feldt	Lower-bound	
Factor	1,000	1,000	

Tests the null hypothesis that the error covariance matrix of the orthonormalized transformed dependent variables is proportional to an identity matrix.a	
a. Design: Intercept + Harm 
 Within Subjects Design: Factor	
b. May be used to adjust the degrees of freedom for the averaged tests of significance. Corrected tests are displayed in the Tests of Within-Subjects Effects table.	


Tests of Within-Subjects Effects	
Measure:   MEASURE_1  	
Source	Type III Sum of Squares	df	Mean Square	F	
Factor	Sphericity Assumed	991,000	1	991,000	830,650	
	Greenhouse-Geisser	991,000	1,000	991,000	830,650	
	Huynh-Feldt	991,000	1,000	991,000	830,650	
	Lower-bound	991,000	1,000	991,000	830,650	
Factor * Harm	Sphericity Assumed	59,400	1	59,400	49,789	
	Greenhouse-Geisser	59,400	1,000	59,400	49,789	
	Huynh-Feldt	59,400	1,000	59,400	49,789	
	Lower-bound	59,400	1,000	59,400	49,789	
Error(Factor)	Sphericity Assumed	21,475	18	1,193		
	Greenhouse-Geisser	21,475	18,000	1,193		
	Huynh-Feldt	21,475	18,000	1,193		
	Lower-bound	21,475	18,000	1,193		

Tests of Within-Subjects Effects	
Measure:   MEASURE_1  	
Source	Sig.	Partial Eta Squared	
Factor	Sphericity Assumed	,000	,979	
	Greenhouse-Geisser	,000	,979	
	Huynh-Feldt	,000	,979	
	Lower-bound	,000	,979	
Factor * Harm	Sphericity Assumed	,000	,734	
	Greenhouse-Geisser	,000	,734	
	Huynh-Feldt	,000	,734	
	Lower-bound	,000	,734	
Error(Factor)	Sphericity Assumed			
	Greenhouse-Geisser			
	Huynh-Feldt			
	Lower-bound			


Tests of Within-Subjects Contrasts	
Measure:   MEASURE_1  	
Source	Factor	Type III Sum of Squares	df	Mean Square	F	Sig.	
Factor	Level 2 vs. Level 1	1982,001	1	1982,001	830,650	,000	
Factor * Harm	Level 2 vs. Level 1	118,801	1	118,801	49,789	,000	
Error(Factor)	Level 2 vs. Level 1	42,949	18	2,386			

Tests of Within-Subjects Contrasts	
Measure:   MEASURE_1  	
Source	Factor	Partial Eta Squared	
Factor	Level 2 vs. Level 1	,979	
Factor * Harm	Level 2 vs. Level 1	,734	
Error(Factor)	Level 2 vs. Level 1		


Tests of Between-Subjects Effects	
Measure:   MEASURE_1  	
Transformed Variable:   Average  	
Source	Type III Sum of Squares	df	Mean Square	F	Sig.	Partial Eta Squared	
Intercept	4565,173	1	4565,173	1200,227	,000	,985	
Harm	39,173	1	39,173	10,299	,005	,364	
Error	68,465	18	3,804				


Custom Hypothesis Tests


Contrast Results (K Matrix)	
Harm Simple Contrasta	Averaged Variable	
	MEASURE_1	
Level 2 vs. Level 1	Contrast Estimate	-2,813	
	Hypothesized Value	0	
	Difference (Estimate - Hypothesized)	-2,813	
	Std. Error	,877	
	Sig.	,005	
	95% Confidence Interval for Difference	Lower Bound	-4,655	
		Upper Bound	-,971	

a. Reference category = 1	


Test Results	
Measure:   MEASURE_1  	
Transformed Variable:   AVERAGE  	
Source	Sum of Squares	df	Mean Square	F	Sig.	Partial Eta Squared	
Contrast	39,173	1	39,173	10,299	,005	,364	
Error	68,465	18	3,804				

CROSSTABS
  /TABLES=Harm BY AMPD
  /FORMAT=AVALUE TABLES
  /STATISTICS=CHISQ RISK
  /CELLS=COUNT ROW
  /COUNT ROUND CELL.


Crosstabs


Case Processing Summary	
	Cases	
	Valid	Missing	Total	
	N	Percent	N	Percent	N	Percent	
Harm * SPD diagnosis at T8	20	83,3%	4	16,7%	24	100,0%	


Harm * SPD diagnosis at T8 Crosstabulation	
	SPD diagnosis at T8	Total	
	Diagnosis	Remission		
Harm	Experimental Group	Count	2	9	11	
		% within Harm	18,2%	81,8%	100,0%	
	Control Group	Count	5	4	9	
		% within Harm	55,6%	44,4%	100,0%	
Total	Count	7	13	20	
	% within Harm	35,0%	65,0%	100,0%	


Chi-Square Tests	
	Value	df	Asymptotic Significance (2-sided)	Exact Sig. (2-sided)	Exact Sig. (1-sided)	
Pearson Chi-Square	3,039a	1	,081			
Continuity Correctionb	1,618	1	,203			
Likelihood Ratio	3,101	1	,078			
Fisher's Exact Test				,160	,102	
Linear-by-Linear Association	2,887	1	,089			
N of Valid Cases	20					

a. 2 cells (50,0%) have expected count less than 5. The minimum expected count is 3,15.	
b. Computed only for a 2x2 table	


Risk Estimate	
	Value	95% Confidence Interval	
		Lower	Upper	
Odds Ratio for Harm (Experimental Group / Control Group)	,178	,024	1,339	
For cohort SPD diagnosis at T8 = Diagnosis	,327	,082	1,305	
For cohort SPD diagnosis at T8 = Remission	1,841	,842	4,023	
N of Valid Cases	20			

CROSSTABS
  /TABLES=Harm BY Dropouts
  /FORMAT=AVALUE TABLES
  /STATISTICS=CHISQ RISK
  /CELLS=COUNT ROW
  /COUNT ROUND CELL.


Crosstabs

Case Processing Summary	
	Cases	
	Valid	Missing	Total	
	N	Percent	N	Percent	N	Percent	
Harm * Dropouts at T8	24	100,0%	0	0,0%	24	100,0%	


Harm * Dropouts at T8 Crosstabulation	
	Dropouts at T8	Total	
	Completed	Dropout		
Harm	Experimental Group	Count	11	1	12	
		% within Harm	91,7%	8,3%	100,0%	
	Control Group	Count	9	3	12	
		% within Harm	75,0%	25,0%	100,0%	
Total	Count	20	4	24	
	% within Harm	83,3%	16,7%	100,0%	


Chi-Square Tests	
	Value	df	Asymptotic Significance (2-sided)	Exact Sig. (2-sided)	Exact Sig. (1-sided)	
Pearson Chi-Square	1,200a	1	,273			
Continuity Correctionb	,300	1	,584			
Likelihood Ratio	1,247	1	,264			
Fisher's Exact Test				,590	,295	
Linear-by-Linear Association	1,150	1	,284			
N of Valid Cases	24					

a. 2 cells (50,0%) have expected count less than 5. The minimum expected count is 2,00.	
b. Computed only for a 2x2 table	


Risk Estimate	
	Value	95% Confidence Interval	
		Lower	Upper	
Odds Ratio for Harm (Experimental Group / Control Group)	3,667	,323	41,590	
For cohort Dropouts at T8 = Completed	1,222	,845	1,767	
For cohort Dropouts at T8 = Dropout	,333	,040	2,769	
N of Valid Cases	24			


Oneway

ANOVA	
Pre-assessment  	
	Sum of Squares	df	Mean Square	F	Sig.	
Between Groups	24,000	1	24,000	,474	,498	
Within Groups	1113,833	22	50,629			
Total	1137,833	23				

ONEWAY GSIT2 BY Harm
  /MISSING ANALYSIS.


Oneway


ANOVA	
SCL90R Total score at T2  	
	Sum of Squares	df	Mean Square	F	Sig.	
Between Groups	104,167	1	104,167	1,228	,280	
Within Groups	1865,667	22	84,803			
Total	1969,833	23				

ONEWAY MASAT2 BY Harm
  /MISSING ANALYSIS.


Oneway

ANOVA	
MAS-A Total score at T2  	
	Sum of Squares	df	Mean Square	F	Sig.	
Between Groups	3,375	1	3,375	,446	,511	
Within Groups	166,583	22	7,572			
Total	169,958	23				

ONEWAY Age BY Harm
  /MISSING ANALYSIS.


Oneway

ANOVA	
Age  	
	Sum of Squares	df	Mean Square	F	Sig.	
Between Groups	2,667	1	2,667	,233	,634	
Within Groups	251,833	22	11,447			
Total	254,500	23				

ONEWAY Education BY Harm
  /MISSING ANALYSIS.


Oneway


ANOVA	
Education  	
	Sum of Squares	df	Mean Square	F	Sig.	
Between Groups	,042	1	,042	,067	,797	
Within Groups	13,583	22	,617			
Total	13,625	23				

ONEWAY Income BY Harm
  /MISSING ANALYSIS.


Oneway


ANOVA	
Annual family income  	
	Sum of Squares	df	Mean Square	F	Sig.	
Between Groups	,000	1	,000	,000	1,000	
Within Groups	11,333	22	,515			
Total	11,333	23				

CROSSTABS
  /TABLES=Harm BY Sex
  /FORMAT=AVALUE TABLES
  /STATISTICS=CHISQ RISK
  /CELLS=COUNT ROW
  /COUNT ROUND CELL.


Crosstabs


Case Processing Summary	
	Cases	
	Valid	Missing	Total	
	N	Percent	N	Percent	N	Percent	
Harm * Sex	24	100,0%	0	0,0%	24	100,0%	


Harm * Sex Crosstabulation	
	Sex	Total	
	Male	Female		
Harm	Experimental Group	Count	7	5	12	
		% within Harm	58,3%	41,7%	100,0%	
	Control Group	Count	8	4	12	
		% within Harm	66,7%	33,3%	100,0%	
Total	Count	15	9	24	
	% within Harm	62,5%	37,5%	100,0%	


Chi-Square Tests	
	Value	df	Asymptotic Significance (2-sided)	Exact Sig. (2-sided)	Exact Sig. (1-sided)	
Pearson Chi-Square	,178a	1	,673			
Continuity Correctionb	,000	1	1,000			
Likelihood Ratio	,178	1	,673			
Fisher's Exact Test				1,000	,500	
Linear-by-Linear Association	,170	1	,680			
N of Valid Cases	24					

a. 2 cells (50,0%) have expected count less than 5. The minimum expected count is 4,50.	
b. Computed only for a 2x2 table	


Risk Estimate	
	Value	95% Confidence Interval	
		Lower	Upper	
Odds Ratio for Harm (Experimental Group / Control Group)	,700	,133	3,684	
For cohort Sex = Male	,875	,469	1,632	
For cohort Sex = Female	1,250	,440	3,548	
N of Valid Cases	24			


General Linear Model


Within-Subjects Factors	
Measure:   MEASURE_1  	
factor1	Dependent Variable	
1	T1	
2	T9	


Multivariate Testsa	
Effect	Value	F	Hypothesis df	Error df	Sig.	
factor1	Pillai's Trace	,893	158,070b	1,000	19,000	,000	
	Wilks' Lambda	,107	158,070b	1,000	19,000	,000	
	Hotelling's Trace	8,319	158,070b	1,000	19,000	,000	
	Roy's Largest Root	8,319	158,070b	1,000	19,000	,000	

Multivariate Testsa	
Effect	Partial Eta Squared	
factor1	Pillai's Trace	,893	
	Wilks' Lambda	,893	
	Hotelling's Trace	,893	
	Roy's Largest Root	,893	

a. Design: Intercept 
 Within Subjects Design: factor1	
b. Exact statistic	


Mauchly's Test of Sphericitya	
Measure:   MEASURE_1  	
Within Subjects Effect	Mauchly's W	Approx. Chi-Square	df	Sig.	Epsilonb	
					Greenhouse-Geisser	
factor1	1,000	,000	0	.	1,000	

Mauchly's Test of Sphericitya	
Measure:   MEASURE_1  	
Within Subjects Effect	Epsilon	
	Huynh-Feldt	Lower-bound	
factor1	1,000	1,000	

Tests the null hypothesis that the error covariance matrix of the orthonormalized transformed dependent variables is proportional to an identity matrix.a	
a. Design: Intercept 
 Within Subjects Design: factor1	
b. May be used to adjust the degrees of freedom for the averaged tests of significance. Corrected tests are displayed in the Tests of Within-Subjects Effects table.	


Tests of Within-Subjects Effects	
Measure:   MEASURE_1  	
Source	Type III Sum of Squares	df	Mean Square	F	Sig.	
factor1	Sphericity Assumed	6002,500	1	6002,500	158,070	,000	
	Greenhouse-Geisser	6002,500	1,000	6002,500	158,070	,000	
	Huynh-Feldt	6002,500	1,000	6002,500	158,070	,000	
	Lower-bound	6002,500	1,000	6002,500	158,070	,000	
Error(factor1)	Sphericity Assumed	721,500	19	37,974			
	Greenhouse-Geisser	721,500	19,000	37,974			
	Huynh-Feldt	721,500	19,000	37,974			
	Lower-bound	721,500	19,000	37,974			

Tests of Within-Subjects Effects	
Measure:   MEASURE_1  	
Source	Partial Eta Squared	
factor1	Sphericity Assumed	,893	
	Greenhouse-Geisser	,893	
	Huynh-Feldt	,893	
	Lower-bound	,893	
Error(factor1)	Sphericity Assumed		
	Greenhouse-Geisser		
	Huynh-Feldt		
	Lower-bound		


Tests of Within-Subjects Contrasts	
Measure:   MEASURE_1  	
Source	factor1	Type III Sum of Squares	df	Mean Square	F	Sig.	
factor1	Linear	6002,500	1	6002,500	158,070	,000	
Error(factor1)	Linear	721,500	19	37,974			

Tests of Within-Subjects Contrasts	
Measure:   MEASURE_1  	
Source	factor1	Partial Eta Squared	
factor1	Linear	,893	
Error(factor1)	Linear		


Tests of Between-Subjects Effects	
Measure:   MEASURE_1  	
Transformed Variable:   Average  	
Source	Type III Sum of Squares	df	Mean Square	F	Sig.	Partial Eta Squared	
Intercept	64000,000	1	64000,000	827,211	,000	,978	
Error	1470,000	19	77,368				

GLM GSIT2 GSIT8
  /WSFACTOR=factor1 2 Polynomial
  /METHOD=SSTYPE(3)
  /PRINT=ETASQ
  /CRITERIA=ALPHA(.05)
  /WSDESIGN=factor1.


General Linear Model


Within-Subjects Factors	
Measure:   MEASURE_1  	
factor1	Dependent Variable	
1	GSIT2	
2	GSIT8	


Multivariate Testsa	
Effect	Value	F	Hypothesis df	Error df	Sig.	
factor1	Pillai's Trace	,976	772,366b	1,000	19,000	,000	
	Wilks' Lambda	,024	772,366b	1,000	19,000	,000	
	Hotelling's Trace	40,651	772,366b	1,000	19,000	,000	
	Roy's Largest Root	40,651	772,366b	1,000	19,000	,000	

Multivariate Testsa	
Effect	Partial Eta Squared	
factor1	Pillai's Trace	,976	
	Wilks' Lambda	,976	
	Hotelling's Trace	,976	
	Roy's Largest Root	,976	

a. Design: Intercept 
 Within Subjects Design: factor1	
b. Exact statistic	


Mauchly's Test of Sphericitya	
Measure:   MEASURE_1  	
Within Subjects Effect	Mauchly's W	Approx. Chi-Square	df	Sig.	Epsilonb	
					Greenhouse-Geisser	
factor1	1,000	,000	0	.	1,000	

Mauchly's Test of Sphericitya	
Measure:   MEASURE_1  	
Within Subjects Effect	Epsilon	
	Huynh-Feldt	Lower-bound	
factor1	1,000	1,000	

Tests the null hypothesis that the error covariance matrix of the orthonormalized transformed dependent variables is proportional to an identity matrix.a	
a. Design: Intercept 
 Within Subjects Design: factor1	
b. May be used to adjust the degrees of freedom for the averaged tests of significance. Corrected tests are displayed in the Tests of Within-Subjects Effects table.	


Tests of Within-Subjects Effects	
Measure:   MEASURE_1  	
Source	Type III Sum of Squares	df	Mean Square	F	Sig.	
factor1	Sphericity Assumed	37699,600	1	37699,600	772,366	,000	
	Greenhouse-Geisser	37699,600	1,000	37699,600	772,366	,000	
	Huynh-Feldt	37699,600	1,000	37699,600	772,366	,000	
	Lower-bound	37699,600	1,000	37699,600	772,366	,000	
Error(factor1)	Sphericity Assumed	927,400	19	48,811			
	Greenhouse-Geisser	927,400	19,000	48,811			
	Huynh-Feldt	927,400	19,000	48,811			
	Lower-bound	927,400	19,000	48,811			

Tests of Within-Subjects Effects	
Measure:   MEASURE_1  	
Source	Partial Eta Squared	
factor1	Sphericity Assumed	,976	
	Greenhouse-Geisser	,976	
	Huynh-Feldt	,976	
	Lower-bound	,976	
Error(factor1)	Sphericity Assumed		
	Greenhouse-Geisser		
	Huynh-Feldt		
	Lower-bound		


Tests of Within-Subjects Contrasts	
Measure:   MEASURE_1  	
Source	factor1	Type III Sum of Squares	df	Mean Square	F	Sig.	
factor1	Linear	37699,600	1	37699,600	772,366	,000	
Error(factor1)	Linear	927,400	19	48,811			

Tests of Within-Subjects Contrasts	
Measure:   MEASURE_1  	
Source	factor1	Partial Eta Squared	
factor1	Linear	,976	
Error(factor1)	Linear		


Tests of Between-Subjects Effects	
Measure:   MEASURE_1  	
Transformed Variable:   Average  	
Source	Type III Sum of Squares	df	Mean Square	F	Sig.	Partial Eta Squared	
Intercept	265690,000	1	265690,000	2262,712	,000	,992	
Error	2231,000	19	117,421				

GLM MASAT2 MASAT8
  /WSFACTOR=factor1 2 Polynomial
  /METHOD=SSTYPE(3)
  /PRINT=ETASQ
  /CRITERIA=ALPHA(.05)
  /WSDESIGN=factor1.


General Linear Model


Within-Subjects Factors	
Measure:   MEASURE_1  	
factor1	Dependent Variable	
1	MASAT2	
2	MASAT8	


Multivariate Testsa	
Effect	Value	F	Hypothesis df	Error df	Sig.	
factor1	Pillai's Trace	,929	246,824b	1,000	19,000	,000	
	Wilks' Lambda	,071	246,824b	1,000	19,000	,000	
	Hotelling's Trace	12,991	246,824b	1,000	19,000	,000	
	Roy's Largest Root	12,991	246,824b	1,000	19,000	,000	

Multivariate Testsa	
Effect	Partial Eta Squared	
factor1	Pillai's Trace	,929	
	Wilks' Lambda	,929	
	Hotelling's Trace	,929	
	Roy's Largest Root	,929	

a. Design: Intercept 
 Within Subjects Design: factor1	
b. Exact statistic	


Mauchly's Test of Sphericitya	
Measure:   MEASURE_1  	
Within Subjects Effect	Mauchly's W	Approx. Chi-Square	df	Sig.	Epsilonb	
					Greenhouse-Geisser	
factor1	1,000	,000	0	.	1,000	

Mauchly's Test of Sphericitya	
Measure:   MEASURE_1  	
Within Subjects Effect	Epsilon	
	Huynh-Feldt	Lower-bound	
factor1	1,000	1,000	

Tests the null hypothesis that the error covariance matrix of the orthonormalized transformed dependent variables is proportional to an identity matrix.a	
a. Design: Intercept 
 Within Subjects Design: factor1	
b. May be used to adjust the degrees of freedom for the averaged tests of significance. Corrected tests are displayed in the Tests of Within-Subjects Effects table.	


Tests of Within-Subjects Effects	
Measure:   MEASURE_1  	
Source	Type III Sum of Squares	df	Mean Square	F	Sig.	
factor1	Sphericity Assumed	1050,625	1	1050,625	246,824	,000	
	Greenhouse-Geisser	1050,625	1,000	1050,625	246,824	,000	
	Huynh-Feldt	1050,625	1,000	1050,625	246,824	,000	
	Lower-bound	1050,625	1,000	1050,625	246,824	,000	
Error(factor1)	Sphericity Assumed	80,875	19	4,257			
	Greenhouse-Geisser	80,875	19,000	4,257			
	Huynh-Feldt	80,875	19,000	4,257			
	Lower-bound	80,875	19,000	4,257			

Tests of Within-Subjects Effects	
Measure:   MEASURE_1  	
Source	Partial Eta Squared	
factor1	Sphericity Assumed	,929	
	Greenhouse-Geisser	,929	
	Huynh-Feldt	,929	
	Lower-bound	,929	
Error(factor1)	Sphericity Assumed		
	Greenhouse-Geisser		
	Huynh-Feldt		
	Lower-bound		


Tests of Within-Subjects Contrasts	
Measure:   MEASURE_1  	
Source	factor1	Type III Sum of Squares	df	Mean Square	F	Sig.	
factor1	Linear	1050,625	1	1050,625	246,824	,000	
Error(factor1)	Linear	80,875	19	4,257			

Tests of Within-Subjects Contrasts	
Measure:   MEASURE_1  	
Source	factor1	Partial Eta Squared	
factor1	Linear	,929	
Error(factor1)	Linear		


Tests of Between-Subjects Effects	
Measure:   MEASURE_1  	
Transformed Variable:   Average  	
Source	Type III Sum of Squares	df	Mean Square	F	Sig.	Partial Eta Squared	
Intercept	9394,225	1	9394,225	829,127	,000	,978	
Error	215,275	19	11,330				
